# Integrative Bulk and Single-Cell Transcriptome Profiling of Telomere-Related Genes Reveals a Robust Prognostic Signature and Immunotherapeutic Landscape in Neuroblastoma

**DOI:** 10.7150/jca.129718

**Published:** 2026-05-18

**Authors:** Yeerfan Aierken, Lulu Zheng, Tao Liu, Kezhe Tan, Zhibao Lv

**Affiliations:** Department of General Surgery, Shanghai Children's Hospital, School of Medicine, Shanghai Jiao Tong University, Shanghai, 200040, China

**Keywords:** neuroblastoma, telomere-related genes (TRGs), prognostic model, tumor immune microenvironment, *PSAT1*

## Abstract

**Purpose:**

Neuroblastoma (NB) is the most common extracranial solid tumor in children with poor overall survival. Increasing evidence indicates that telomeres contribute to tumorigenesis and influence cancer prognosis. However, the biological and clinical implications of telomere-related genes (TRGs) in NB remain poorly defined.

**Materials and Methods:**

We integrated data from multiple independent cohorts to elucidate the roles of TRGs in NB. Differential expression and weighted gene co-expression network analyses (WGCNA) were performed to identify telomere-related differentially expressed genes (TRDEGs) linked to patient survival. Consensus clustering based on TRDEG expression patterns was conducted to stratify molecular subtypes, followed by functional enrichment analysis. A prognostic signature was then built using machine-learning algorithms to predict clinical outcomes and potential therapeutic responses. Single-cell RNA sequencing (scRNA-seq) data were used for signature gene expression validation and to guide functional candidate selection. Quantitative RT-PCR was performed to verify the TRDEG signature, and functional assays were performed to explore the role of *PSAT1* in NB progression.

**Results:**

First, we identified 103 telomere-related differentially expressed genes (TRDEGs) significantly linked to NB patient survival. Consensus clustering of TRDEGs revealed two NB molecular subtypes with distinct biological processes and clinical outcomes. We established an eight-gene prognostic signature (*ARHGAP23, CHD5, E2F3, ELOVL6, FEN1, GMPS, LRR1,* and *PSAT1*) that demonstrated high predictive accuracy, with 1-, 3-, and 5-year survival AUCs of 0.885, 0.903, and 0.911, respectively. The model showed consistent robustness across validation cohorts. Multivariate Cox regression confirmed the risk score as an independent prognostic factor. Integrating the risk score with clinical parameters within a nomogram yielded superior prognostic performance compared with traditional stratification schemes. High-risk patients showed decreased immune cell infiltration and increased immune evasion patterns, corresponding to poorer immunotherapy response. Distinct chemosensitivity profiles characterized the two risk groups. Quantitative RT-PCR validated the TRDEG signature. Last, *PSAT1* was identified as a representative gene within the TRDEG signature through integration of scRNA-seq data, exhibited tumor-cell-specific expression, and was experimentally confirmed to promote NB cell proliferation, inhibit apoptosis, and enhance migratory capacity.

**Conclusion:**

TRGs play a pivotal role in shaping NB prognosis and treatment response. The validated TRDEG signature provides a foundation for individual risk assessment and future development of precision therapies and immunotherapeutic strategies. Among these genes, *PSAT1* emerges as a key oncogenic driver.

## Introduction

Neuroblastoma (NB) is the predominant extracranial solid tumor in pediatric populations, responsible for an estimated 8-10% of all childhood cancers and contributing to 15% of cancer-related mortality in children 1. Current risk stratification, as established by the Children's Oncology Group (COG), classifies NB patients into very low-, low-, intermediate-, and high-risk categories using criteria including age, the International Neuroblastoma Staging System (INSS), histopathological findings, DNA copy number variations, *MYCN* amplification status, and other clinical characteristics. Despite advances in treatment, the overall survival (OS) rate for high-risk NB remains dismal at under 50%, underscoring the urgent need to enhance treatment outcomes and quality of life for these patients 2. Although genetic alterations in genes including *MYCN, ALK, TERT, PHOX2B*, and *ATRX* are recognized as important risk factors, high-risk neuroblastoma exhibits considerable genetic heterogeneity. Notably, more than 25% of these patients lack the aforementioned aberrations 3. Consequently, discovering novel biomarkers is imperative to improve prognostic accuracy and current clinical evaluation system.

Telomeres, composed of repetitive TTAGGG sequences at eukaryotic chromosome ends, play critical roles in regulating cellular lifespan and division potential. In healthy human cells, telomere length undergoes progressive shortening with each cell division 4. This attrition is directly linked to several pathologies, including tumorigenesis 5, 6. Nevertheless, the role of telomere shortening in cancer is complex and controversial, as it can function as a tumor suppressor by inhibiting cell proliferation, yet also promote genomic instability, thereby increasing the risk of tumor development 7. Numerous researches have explored the potential applications of telomeres and telomere-related genes (TRGs) as biomarkers for glioma, breast cancer, bladder cancer, and pancreatic cancer 8-12. In NB, emerging evidence indicated that heritable extensions in leukocyte telomere length confer susceptibility to its pathogenesis 13. Moreover, the acquisition of a telomere maintenance mechanisms (TMMs) is a fundamental hallmark of high-risk NB 14. This occurs primarily through two mutually exclusive pathways: telomerase activation, commonly driven by *MYCN* amplification or TERT rearrangements, or the alternative lengthening of telomeres (ALT) pathway, which is strongly associated with *ATRX* mutations 15-17. Importantly, the presence of either TMMs, and particularly the ALT phenotype, is linked to aggressive disease and poorer patient outcomes. The biological understanding of these mechanisms has revealed profound therapeutic insights; for instance, ALT-positive cells exhibit constitutive ataxia-telangiectasia mutated (ATM) activation due to chronic telomere dysfunction, which confers resistance to conventional chemotherapy but also creates a unique vulnerability to ATM inhibition 18. Additionally, epigenetic mechanisms such as METTL3-mediated m6A modification of TERRA lncRNA contribute to ALT, highlighting further therapeutic opportunities 19. Despite these insights, prior research has predominantly focused on telomere length and stability in cancer prognosis, leaving the specific roles of TRGs in NB inadequately explored.

This study aimed to develop an innovative risk model based on TRGs to predict clinical outcomes in NB, while also evaluating its potential implications in tumor immune evasion and the therapeutic drug selection. Through analyzing single-cell RNA sequencing (scRNA-seq) data, we examined the expression profiles of these model genes across multiple cell types, strongly implicating *PSAT1* in malignant cells. Functional cell experiments confirmed *PSAT1*'s critical role in driving NB progression. Together, our results contribute not only to the identification of novel prognostic biomarkers and the advancement of TRG research in NB, but also provided essential insights for early diagnosis, optimization of treatment strategies, and enhanced management of prognosis in NB.

## Materials and Methods

### Acquisition of TRGs and transcriptome data

The workflow of this study is illustrated in **Figure S1**. We obtained RNA expression profiles and associated clinical annotations from multiple cohorts, including GSE62564, GSE16476, GSE85047, E-MTAB-8248, E-TABM-38 and EGAS00001001308 (abbreviated as EGAS), from the Gene Expression Omnibus (GEO) and the R2 platform (https://hgserver1.amc.nl/cgi-bin/r2/main.cgi). GSE62564, which had the largest sample size and the most comprehensive clinical as well as survival information, was designated as the training cohort, while the remaining datasets were used for validation purposes. We also analyzed three immunotherapy cohorts with varying treatment responses, which were downloaded from the TIGER portal (http://tiger.canceromics.org/). These datasets included GSE91061 (anti-PD-1), phs000452 (anti-PD-1), and RCC-Braun_2020 (anti-PD-1 and EVEROLIMUS). The data from GEO were processed with log2(x) transformation to acquire samples with normalized RNA-seq or microarray data, batch effects were assessed and corrected using the “sva” package in R software. The data from R2 were pre-processed according to the platform's guidelines to ensure consistency and comparability across datasets.

The TelNet database, accessible at http://www.cancertelsys.org/telnet/, compiles data on telomere-associated genes, cataloging approximately 2,000 human genes and over 1,100 yeast genes. These genes include annotations on maintenance mechanisms, functional roles, homologs, and significance scores related to telomere biology 20. From this resource, we extracted 2,089 human genes linked to telomere functions (**Table S1**).

### Analysis of scRNA-seq data

NB scRNA-seq data were acquired from the GEO repository under accession number GSE220946. The dataset comprised 15 samples, collectively containing 38,026 cells (2,535 cells per sample). Preprocessing and quality control were performed applying the “Seurat” R package. Low-quality cells and genes were filtered using the following thresholds: 1) Cells expressing <500 or >9,500 genes excluded; 2) Genes detected in <3 cells excluded; 3) Cells retaining ≥3% red blood cell genes excluded. Following filtration, 34,697 high-quality cells were retained. Gene expression data were processed with the NormalizeData method to achieve normalization, after which highly variable features were selected. Dimensionality reduction was performed by applying principal component analysis (PCA), and the first 20 principal components were retained for subsequent analyses. The Harmony algorithm was employed to correct for technical batch effects between samples. Using the FindClusters function at a resolution of 0.5, we identified 23 cell clusters. Two-dimensional visualization was achieved via uniform manifold approximation and projection (UMAP). Cluster-specific marker genes were determined by employing the FindAllMarkers function with normalized counts. Finally, clusters were annotated using specific biomarkers for each cell type, enabling quantification and evaluation of cell type proportions (**Table S2**).

### Weighted gene co-expression network analysis (WGCNA)

To elucidate the co-expression patterns among candidate genes and identify clusters of closely correlated genes, WGCNA was utilized. In this study, the top 5000 genes with the highest variance values were selected and subjected to WGCNA. The soft-thresholding power (β) was determined through the utilization of the PickSoftThreshold function available in the “WGCNA” R package, with an optimal threshold value of 0.9 chosen to guarantee a scale-free network topology. The identification of modules was conducted through hierarchical clustering of the genes. The module eigengenes served as representations of the summarized expression profiles corresponding to each module. These modules were visualized as distinct branches in the dendrogram, each assigned a unique color for differentiation. Furthermore, the associations between individual genes and modules, as well as the interrelationships among modules, were comprehensively investigated.

### Identification of telomere-related differentially expressed genes (TRDEGs) and functional enrichment analysis

In order to pinpoint differentially expressed genes (DEGs) in high-risk NB, we utilized the DESeq2 package to analyze the transcriptomic data from the GSE62564 cohort. By comparing the gene expression profiles between COG high-risk and low-risk tissues, we set a threshold of |log2 fold change| > 0.5 and an adjusted *P* < 0.05 (adjusted via the Benjamini-Hochberg method) to identify significant DEGs, and the results were visualized as volcano plots using the “ggplot2” R package. This approach enabled us to effectively distinguish key genetic markers associated with NB progression.

TRDEGs were identified through intersecting three distinct datasets: 1) DEGs between COG risk-stratified groups, 2) telomere maintenance genes curated in the TelNet database, and 3) module genes derived from WGCNA. Then, through applying the RCircos package, the chromosomal positions of TRDEGs (top 25 genes with P < 0.05) were displayed. Heatmaps were generated to illustrate the top 25 significantly upregulated and downregulated genes (P < 0.05). Functional enrichment was assessed through Gene Ontology (GO) analysis—encompassing biological processes (BP), cellular components (CC), and molecular functions (MF)—and Kyoto Encyclopedia of Genes and Genomes (KEGG) pathways analysis using the “clusterProfiler” package in R. Enrichment results with a* P* < 0.05 were considered statistically significant.

### Construction of subtype analysis according to key TRDEGs

To identify distinct molecular subtypes of NB based on the TRDEGs, consensus clustering was carried out with the “ConsensusClusterPlus” package in R. This approach involved an unsupervised analysis with the partitioning around medoids (PAM) algorithm and Euclidean distance metric to quantify the stability of clustering outcomes across multiple iterations. Specifically, we evaluated potential cluster numbers ranging from k=2 to k=10, repeatedly resampling subsets of the NB cohort at an 80% rate to generate consensus matrices and ensure classification robustness. The optimal number of clusters was determined by analyzing the cumulative distribution function (CDF) to maximize agreement across iterations. Furthermore, PCA was applied to validate the derived molecular subtypes, and Kaplan-Meier survival analysis was performed to assess their clinical significance in relation to patient prognosis. Additionally, we not only employed gene set variation analysis (GSVA) to obtain variations in KEGG pathway enrichment among subtypes but also explored the correlations between these subtypes, presenting the findings through heatmaps.

### Gene set enrichment analysis (GSEA) in telomere-associated subtypes

GSEA, a widely used computational method, assessed the biological functions of gene cohorts by systematically evaluating overlaps with predefined gene sets from established databases. Using the R/Bioconductor package “clusterProfiler”, we computed enrichment score for genes ordered based on their log_2_FC values. This was performed with 1,000 permutations to establish empirical significance, and gene sets were restricted to sizes between 10 and 500 members to ensure robustness. In order to control false discoveries, statistical significance was assessed with Benjamini-Hochberg-adjusted p-values. As the reference database, we employed the specific c2.cp.kegg.v7.5.1.symbols gene set curated from MSigDB.

### Establishment of prognostic risk model according to TRDEGs

To establish a robust prognostic signature reflecting telomere biology in NB, we conducted comprehensive survival analyses. Utilizing gene expression profiles and OS data from a cohort of 498 NB patients, we first performed univariate Cox proportional hazards regression analysis on TRDEGs. This initial screen, conducted using the survival package in R with a significance threshold of *P* < 0.05, identified TRDEGs exhibiting significant associations with patient OS. To enhance the prognostic model and reduce the risk of overfitting, the candidate TRDEGs underwent Least Absolute Shrinkage and Selection Operator (LASSO) Cox regression analysis, which was conducted using the "glmnet" package in R. The optimization of the model was achieved through tenfold cross-validation, which was employed to ascertain the ideal penalization parameter (λ). Specifically, the λ.min value yielding the minimal mean cross-validated error was selected. This process yielded an optimal set of eight key prognostic genes. A prognostic risk score for each patient was then calculated using the linear predictor derived from this LASSO-Cox model: Risk score = Σ (Gene Expressionᵢ × Coefficientᵢ), where Expressionᵢ represents the standardized expression level of each model gene, and Coefficientᵢ denotes its corresponding LASSO-Cox regression coefficient, reflecting the gene's weighted contribution to the prognostic risk.

### Construction of a predictive nomogram

NB patients were categorized into low- and high-risk groups using median LASSO risk score (LRS) from training and validation cohorts. To assess the differences in OS between these groups, Kaplan-Meier survival curves were generated utilizing the “survminer” and “survival” packages in R. Furthermore, the performance of the predictive signature for survival at 1, 3, and 5 years was evaluated through receiver operating characteristic (ROC) curves, which were constructed using the “timeROC” and “ggplot2” packages, with the area under the curve (AUC) metrics serving as the evaluation criteria. Scatter and risk plots were also utilized for Visualization of patient risk distributions within the groups and estimation of death risk. Furthermore, Clinical factors (age, gender, INNS stage, *MYCN* status) were extracted from both training and validation cohorts, summarized in **Table S3**. Each factor's prognostic discriminatory power was calculated via the concordance index (C-index), followed by a bar chart comparing C-index values among datasets. To determine the independent prognostic significance of the risk score for NB OS, we conducted both univariate and multivariate Cox regression analyses, which included the aforementioned clinical factors. A forest plot was generated using the “forestplot” R package. A predictive nomogram along with an associated calibration plot for estimating OS probabilities at 1, 3, and 5 years was constructed utilizing the “rms” package in R. Finally, the clinical applicability of the predictive model was evaluated using decision curve analysis (DCA), which measured true-positive and false-positive rates at incrementally varying clinical decision thresholds.

### The stemness index calculation

For the GSE62564 cohort, the mRNA stemness index (mRNAsi)—a quantitative measure of cellular reprogramming potential—was predicted for each sample using one-class logistic regression. All resulting mRNAsi values were subsequently normalized to a scale ranging from 0 to 1. This normalization allowed for more consistent and interpretable comparisons across samples. Elevated mRNAsi scores are indicative of an increased stem-like phenotype.

### Characterizing the immune infiltration landscape and predicting immunotherapy responses

We assessed the immune cell microenvironment through various algorithms, including the ESTIMATE, EPIC, MCPCOUNTER, QUANTISEQ, and XCELL algorithms, all part of the “IOBR” R package. The results were validated using the single-sample gene set enrichment analysis (ssGSEA) algorithm. We generated group comparison maps using the “ggplot2” R package to illustrate the differences in immune cell expression and immune functions across various groups. Additionally, we calculated the correlation between key genes, TRDEGs risk score, and immune cells using the Spearman algorithm, and we visualized these correlations via heatmaps created with the “ggplot2” package in R.

We further evaluated the TRDEGs risk score's predictive value for immunotherapy response by analyzing key immunological indicators: the tumor immune dysfunction and exclusion (TIDE, available at http://tide.dfci.harvard.edu/) score, cytotoxic T lymphocytes (CTL) score, mRNAsi and expression of *PD-L1*. Utilizing these findings, we computed the telomere-related risk score for patients within the training cohort to explore its role in immunotherapy. Wilcoxon tests were employed to determine the statistical significance of differences in these immunotherapy predictor distributions between patients stratified into high-risk and low-risk categories. Spearman analysis was conducted to explore the relationship between risk score and mRNAsi. The above distribution was visualized using scatter plots, accompanied by its corresponding p-values. In addition, we incorporated multiple immunotherapy cohorts with annotated clinical response data to validate the predictive efficacy of the immunotherapy response. The LASSO-COX algorithm was consistently employed to compute individualized risk scores. Kaplan-Meier survival analysis, implemented via the "survival" R package, was utilized to compare survival outcomes between high- and low-risk groups, with between-group differences assessed using the log-rank test.

### Sensitivity prediction analysis of chemotherapeutic agents

The association between the risk score of TRDEGs and drug sensitivity was predicted utilizing the GDSC and CTRP datasets through the application of the "oncoPredict" R packages. Simultaneously, the "Hmsic" R package was utilized to analyze the correlation between transcriptional profiles and chemotherapeutic sensitivity.

### Cell culture

We acquired the hTERT-immortalized retinal pigmented epithelial cell line (RPE-1)—a commonly used non-NB control—along with the NB cell lines BE(2)-C, SK-N-BE2, IMR-32, SK-N-SH, and SH-SY5Y from the Chinese Academy of Sciences Cell Bank (Shanghai, China). BE(2)-C and RPE-1 cell lines were cultured in Dulbecco's Modified Eagle Medium/Nutrient Mixture F-12 (DMEM/F12; Gibco, #11330032) containing 10% fetal bovine serum (FBS; Sigma-Aldrich, #F8318) and 1% penicillin-streptomycin (Gibco, #15140-122). IMR-32 and SH-SY5Y cells were cultured in a 1:1 mixture of Eagle's Minimum Essential Medium (MEM; BasalMedia, #L510KJ) and Ham's F-12 Nutrient Mix (BasalMedia, #L410KJ), also supplemented with 10% FBS and 1% penicillin-streptomycin. SK-N-BE2 and SK-N-SH cells were cultured in Dulbecco's Modified Eagle Medium (DMEM; Gibco, #11885084) enriched with 10% FBS and 1% penicillin-streptomycin. All cell lines were kept under standard culture conditions at 37°C with 5% CO2 in a cell incubator.

### RNA extraction and quantitative RT-PCR

Total RNA was extracted from cells with TRIzol reagent (#15596018, Invitrigen, Carlsbad, USA), followed by reverse transcription using Evo M-MLV RT Premix (#11706, Accurate biology, Hunan, China). Quantitative PCR analysis was performed using the Hieff UNICON qPCR SYBR Green Master Mix (#11198ES03, YEASEN, Shanghai, China). Relative gene expression was calculated via the 2⁻△△CT method, normalized to *GAPDH*. The sequences of the primers are detailed in **Table S4**.

### Small interfering RNA transfection

Small interfering RNA (siRNA) targeting *PSAT1*, along with a control siRNA, were procured from RIOBIO (Zorin, Shanghai, China). SK-N-BE2 and BE(2)-C cells, which were selected based on the mRNA expression pattern of *PSAT1*, were transfected using ExFect Transfection Reagent (#T101-01, Vazyme, Nanjing, China) according to the manufacturer's instruction. siRNA interference sequences are shown as follows: si-*PSAT1*-1 (sense: 5′-CCGGGCCUCUCUGUAUAAUTT-3′; antisense: 3′-AUUAUACAGAGAGGCCCGGTT-5′), si-*PSAT1*-2 (sense: 5′-GCCGCACUCAGUGUUGUUATT-3′; antisense: 3′-UAACAACACUGAGUGCGGCTT-5′), and nontargeting control siRNA (si-NC, sense: 5′-UUCUCCGAACGUGUCACGUTT-3′; antisense: 3′-ACGUGACACGUUCGGAGAATT-5′). Efficiency of siRNAs were detected by western blot.

### Cell viability assay

Cell viability in NB cell lines was evaluated using the Cell Counting Kit-8 (CCK-8, #K1018, APExBIO, Houston, USA). Cells were seeded into 96-well plates at 5 × 10³ cells per well in 100 µL medium. After incubation, CCK-8 reagent was introduced to achieve a 10% final concentration, and the plates were incubated at 37 °C for 4 hours. Absorbance at 450 nm (OD450) was then measured using a microplate reader., and viability was calculated as a percentage relative to untreated control wells.

### Wound healing assay

NB Cells were plated in 6-well plates and grown to 90% confluence under standard conditions (37°C, 5% CO₂). The cell layer was scratched with a 200-µl pipette suction head after achieving 90% confluence. Then The wells were rinsed twice with PBS to clear cellular debris and replenished with low-serum medium. Precise positioning marks were made adjacent to each wound to ensure consistent imaging fields. Wound areas were photographed at 0 h (baseline) and after a 24 h incubation period using an inverted microscope. Quantification of cell migration into the wound area was performed using ImageJ software.

### Apoptosis measurement by annexin V-FITC/PI flow cytometry

NB cells transfected with siRNAs targeting *PSAT1* (si-*PSAT1*-1 and si-*PSAT1*-2) or si-NC were harvested, washed with PBS. Subsequently, the transfected cells were plated in 6-well plates at a density of 3×10^5^ cells per well and cultured for 48 hours. Apoptosis was quantified with the FITC Annexin V Apoptosis Detection Kit I (#2139225, BD Biosciences, New Jersey, USA) according to the manufacturer's instructions, and data were processed with FlowJo software.

### Western blot

Cellular lysis used RIPA buffer (#P0013B, Beyotime, Shanghai, China), with protein concentrations quantified via BCA Protein Assay Kit (#P002, Beyotime, Shanghai, China). We resolved 30 µg samples per lane on 10% SDS-PAGE gels, transferring proteins to PVDF membranes. After 2-hour blocking in 5% BSA, membranes underwent overnight incubation at 4°C with primary antibodies: anti-PSAT1 (10501-1-AP, Proteintech, Wuhan, China; dilution 1:5000) and anti-HSP90 (13171-1-AP, Proteintech, Wuhan, China; dilution 1:5000). Following three times TBST washes, membranes were incubated with an HRP-conjugated secondary antibody for 1 hour at room temperature, after which protein bands were visualized using an ECL detection system (Bio-Rad, California, USA).

### Statistical analysis

Data analyses and figure generation were conducted using GraphPad Prism (v10.0) and R (v4.4.0), respectively. Pairwise group comparisons employed the Wilcoxon rank-sum test, while differences across multiple groups were assessed using either ANOVA or the Kruskal-Wallis test. Categorical variables underwent chi-square or Fisher's exact testing. For correlation analysis, Spearman's rank correlation method was employed. Survival outcomes were compared using Kaplan-Meier curves with log-rank testing. All experiments included ≥3 biological replicates, with data presented as mean ± standard error of the mean (SEM) in figures. Statistical significance was defined as P < 0.05.

## Results

### Identification of TRDEGs between COG high- and low-risk NB

**Figure 1:**
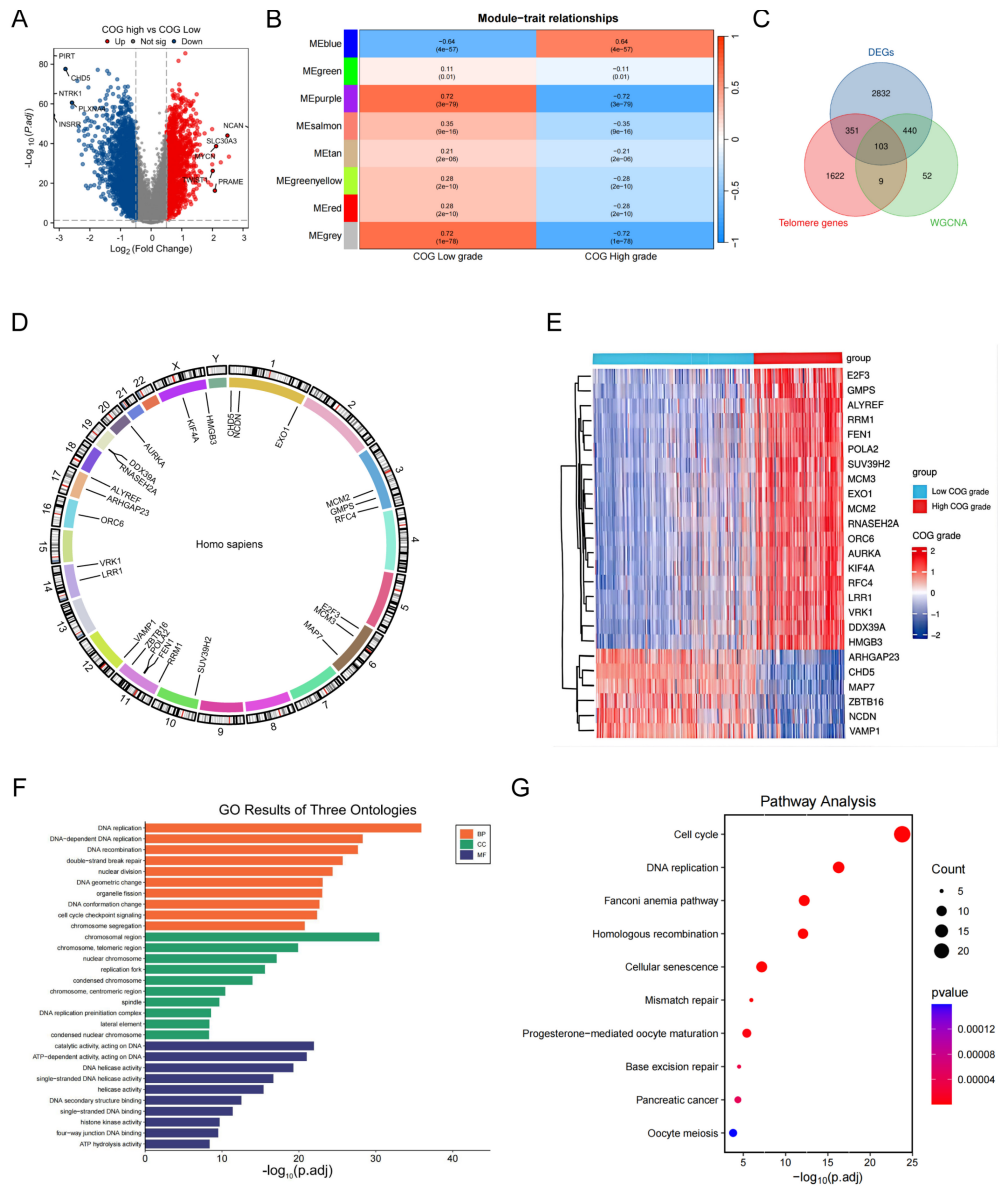
Identification and characterization of TRDEGs in NB. **(A)** Volcano plot showing 3726 DEGs between COG High-and Low-risk groups.** (B)** Heatmap illustrating module-trait relationships identified by WGCNA.** (C)** Overlapping genes among DEGs, TRGs from the TelNet database and WGCNA-derived module genes. **(D)** Chromosome mapping of the top 25 TRDEGs.** (E)** Heatmap of the top 25 TRDEGs.** (F-G)** GO and KEGG enrichment analyses of 103 TRDEGs.

A comprehensive analysis of transcriptomic data from the GSE62564 samples revealed 3726 DEGs (**Table S5**) between COG high-and low-risk NB (a threshold of |log_2_FC| > 0.5 and *P* < 0.05). Among these 3726 DEGs, 1400 were found to be upregulated while 2426 were downregulated in the high-risk COG NB, as illustrated in the volcano plot (**Figure 1A**). As shown in **Figure 1B**, the blue module exhibited a strong positive correlation with COG high risk NB, with the coefficient of 0.64. A total of 103 overlapping genes among DEG, TRGs in the TelNet database, and the module genes highly and positively correlated with COG high risk NB after WGCNA (**Table S6**) were found and deemed as TRDEGs between COG high- and low- risk NB (**Figure 1C) (Table S7**). The chromosome localization map showed that the top 25 TRDEGs (based on adjusted* p*-value) were mainly located on chromosomes 1, 3, 6 and 11, including *CHD5, NCDN* and *EXO1* on chromosome 1; *MCM2, GMPS* and *RFC4* on chromosome 3; *E2F3, MCM3* and *MAP7* on chromosome 6; *RRM1, ZBTB16, FEN1* and *POLA2* were located on chromosome 11 (**Figure 1D**). The top 25 TRDEGs among different COG groups were identified based on the intersection results (**Figure 1E**). GO analysis, which includes biological processes (BP), cellular components (CC) and molecular functions (MF), underscored their significance in “DNA REPLICATION”, “CELL CHECKPOINT SIGNALING PATHWAYS” and “DOUBLE-STRAND BREAK REPAIR” (**Figure 1F**). Furthermore, The KEGG analysis, depicted using a bubble plot, revealed enrichment of these genes in pathways such as “CELL CYCLE”, “CELLULAR SENSCENCE”, “MISMATCH REPAIR”, “HOMOLOGOUS RECOMBINATION” and “DNA REPLICATION” (**Figure 1G**).

### Discovery of telomere-associated subtypes in NB via consensus clustering analysis

**Figure 2:**
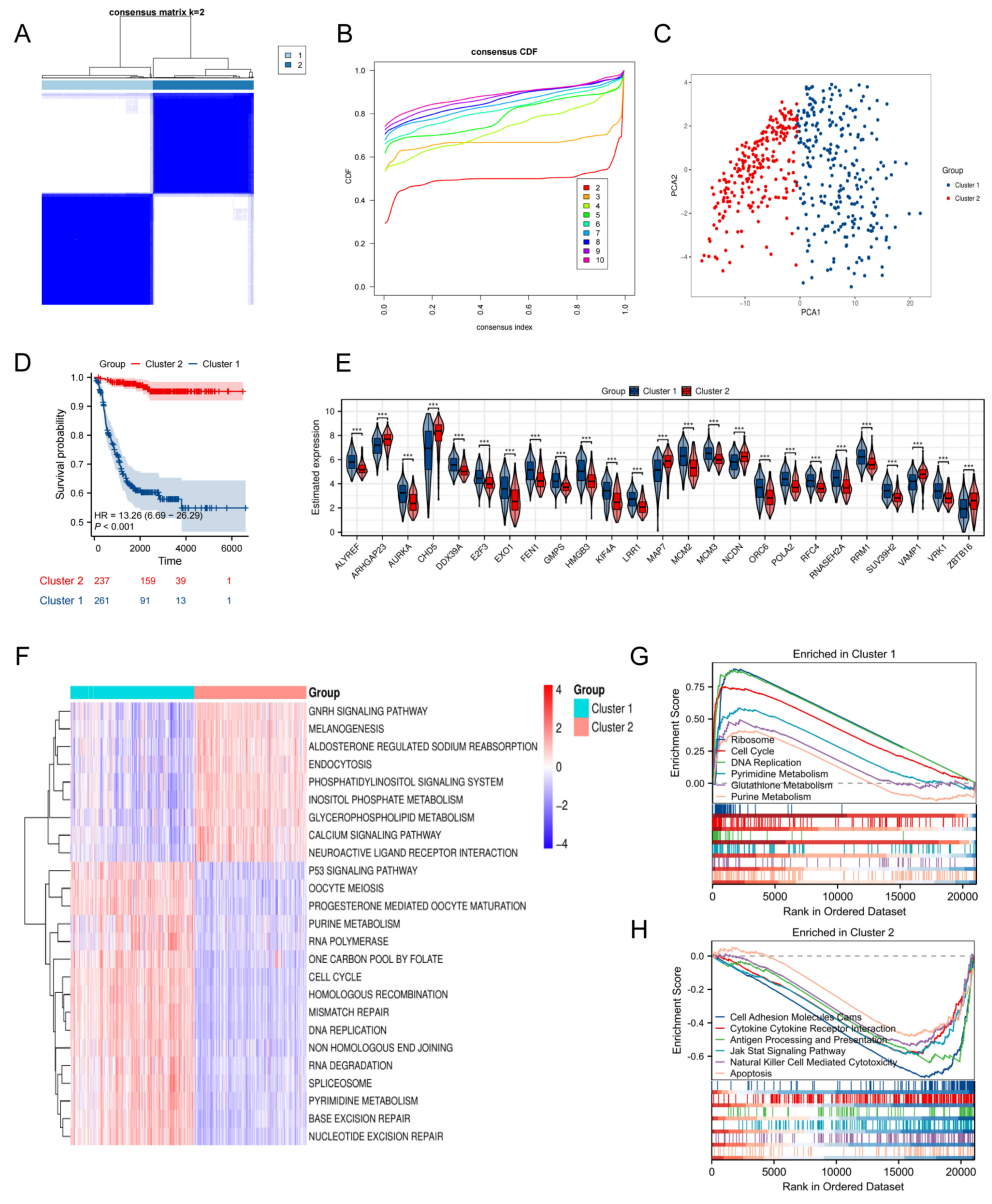
Discovery and functional characterization of TRDEGs-associated molecular subtypes.** (A)** Consensus score matrix of all samples when *k* = 2.** (B)** CDF curves of consensus matrix under *k* = 2-10. **(C)** PCA showing notable variations between two TRDEGs patterns from training cohort. **(D)** Kaplan-Meier curves comparing survival between Cluster 1 and Cluster 2. **(E)** Violin plots showing the expression differences in top 25 TRDEGs among two clusters.** (F)** GSVA showing pathway enrichment differences between Cluster 1 and Cluster 2. **(G-H)** GSEA based on “c2.cp.kegg.v7.5.1.symbols” gene sets showing pathways enriched in Cluster 1 and Cluster 2. *P < 0.05, ** P < 0.01, *** P < 0.001.

Next, we performed consensus clustering on 498 samples from the GSE62564 to investigate the potential telomere-related subtypes in NB. The optimal number of clusters was determined to be 2, based on the area under the curve of the CDF plot (**Figure 2A, B**). A clear separation between these two clusters was evidenced by PCA (**Figure 2C**). Patients in Cluster 2 exhibited significantly better survival outcomes compared to those in Cluster 1, who had relatively poorer prognoses (**Figure 2D**). Additionally, the expression patterns of the top 25 TRDEGs were found to effectively distinguish between the two subtypes (**Figure 2E**).

To explore pathway enrichment patterns distinguishing NB Cluster 1 and Cluster 2, we conducted GSVA (**Table S8**) and GSEA (**Table S9**). GSVA highlighted that Cluster 1 showed marked enrichment in processes such as "CELL CYCLE", "P53 SIGNALING PATHWAY", "ONE CARBON POOL BY FOLATE", and "PYRIMIDINE METABOLISM". In contrast, Cluster 2 was notably enriched in "ENDOCYTOSIS", "CALCIUM SIGNALING PATHWAY", and "GNRH SIGNALING PATHWAY" (**Figure 2F**). Supporting these findings, GSEA further indicated that Cluster 1 was strongly associated with "RIBOSOME", "CELL CYCLE", "DNA REPLICATION", and "PYRIMIDINE METABOLISM", whereas Cluster 2 exhibited dominant enrichment in "APOPTOSIS", "ANTIGEN PROCESSING AND PRESENTATION", and "CYTOKINE-CYTOKINE RECEPTOR INTERACTION" (**Figure 2G, H**). Our integrated results reveal a clear divergence in biological processes between the two clusters—with Cluster 1 enriched in proliferation and metabolism, and Cluster 2 enriched in immune and regulatory functions—underscoring fundamental molecular differences across these NB subtypes.

### Construction and validation of a prognostic model according to TRDEGs

**Figure 3:**
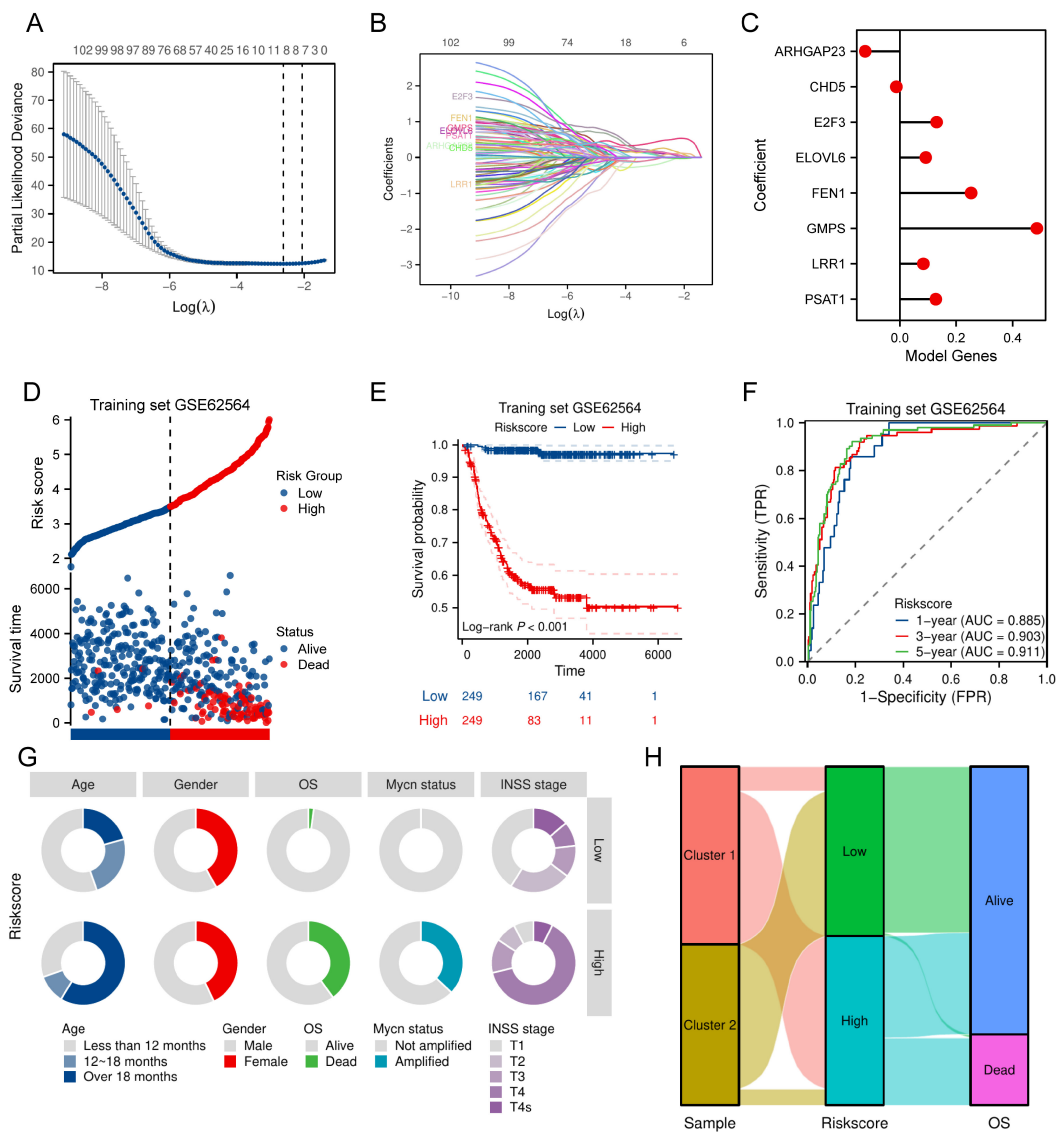
Construction of a prognostic risk model based on key TRDEGs in the training cohort. **(A)** LASSO regression screen in training cohort showing 8 of 103 core genes at the least deviance.** (B)** LASSO regression screen in training cohort showing coefficients of core genes at different λ levels.** (C)** Forest plot showing the coefficients of the eight model genes.** (D)** Distribution of risk scores and survival status of patients in the training cohort.** (E)** Kaplan-Meier curve of training cohort between the two risk groups determined by the 8-gene prognostic risk model.** (F)** Time-dependent ROC curves in training cohort for 8-gene prognostic risk model. **(G)** Distribution of clinical characteristics (age, gender, OS, *MYCN* status, and INSS stage) between the two risk groups. **(H)** Sankey diagram showing the relationships among subclusters, TRDEG-based risk score and survival status.

Univariate Cox regression was first conducted in the training cohort to evaluate the relationship of TRDEGs to survival outcomes. This approach revealed that each of the examined genes demonstrated statistically significant associations with survival outcomes (*P* < 0.05, **Table S10**). Based on these 103 TRDEGs, a refined prognostic signature—designated the TRDEGs signature—was subsequently developed using LASSO regression (**Figure 3A, B**). The LRS was calculated employing coefficients obtained from the LASSO algorithm (**Figure 3C**, LRS = -0.12276 × *ARHGAP23* + -0.01241 × *CHD5* + 0.13063 × *E2F3* + 0.09201 × *ELOVL6* + 0.25257 × *FEN1* + 0.48589 × *GMPS* + 0.08329 × *LRR1* + 0.12765 × *PSAT1*). We then classified NB patients into low- and high-risk groups according to the median LRS. Risk curves and scatter plots revealed clear separation between low- and high-risk groups, demonstrating the risk score's effectiveness in stratifying patients according to survival outcomes (**Figure 3D**). In accordance with this stratification, Kaplan-Meier survival analysis confirmed that individuals in the high-risk group exhibited substantially inferior OS relative to those in the low-risk group within the training cohort (**Figure 3E**). The time-dependent ROC curves were applied to assess the predictive performance of the prognostic signature at 1, 3, and 5 years. The signature displayed robust discriminative capacity, as evidenced by AUC values of 0.885, 0.903, and 0.911 for the respective time points (**Figure 3F**). Extending beyond the training cohort, the signature's generalizability was rigorously evaluated across five independent validation cohorts: GSE16476, GSE85047, EGAS, E-TABM-38, and E-MTAB-8248. Comprehensive analyses within these diverse NB cohorts validated the signature's strong performance as evidenced by risk plots (**Figure S2A-E**), Kaplan-Meier curves (**Figure S2F-J**), and ROC curve analyses (**Figure S2K-O**). Furthermore, A donut chart and Sankey Diagram illustrated the correlation among signature's risk score, telomere-related subcluster and clinical characteristics, suggesting TRDEGs signature's potential utility in clinical prognosis (**Figure 3G, H**).

### Development of the nomogram including the TRDEGs risk score

**Figure 4:**
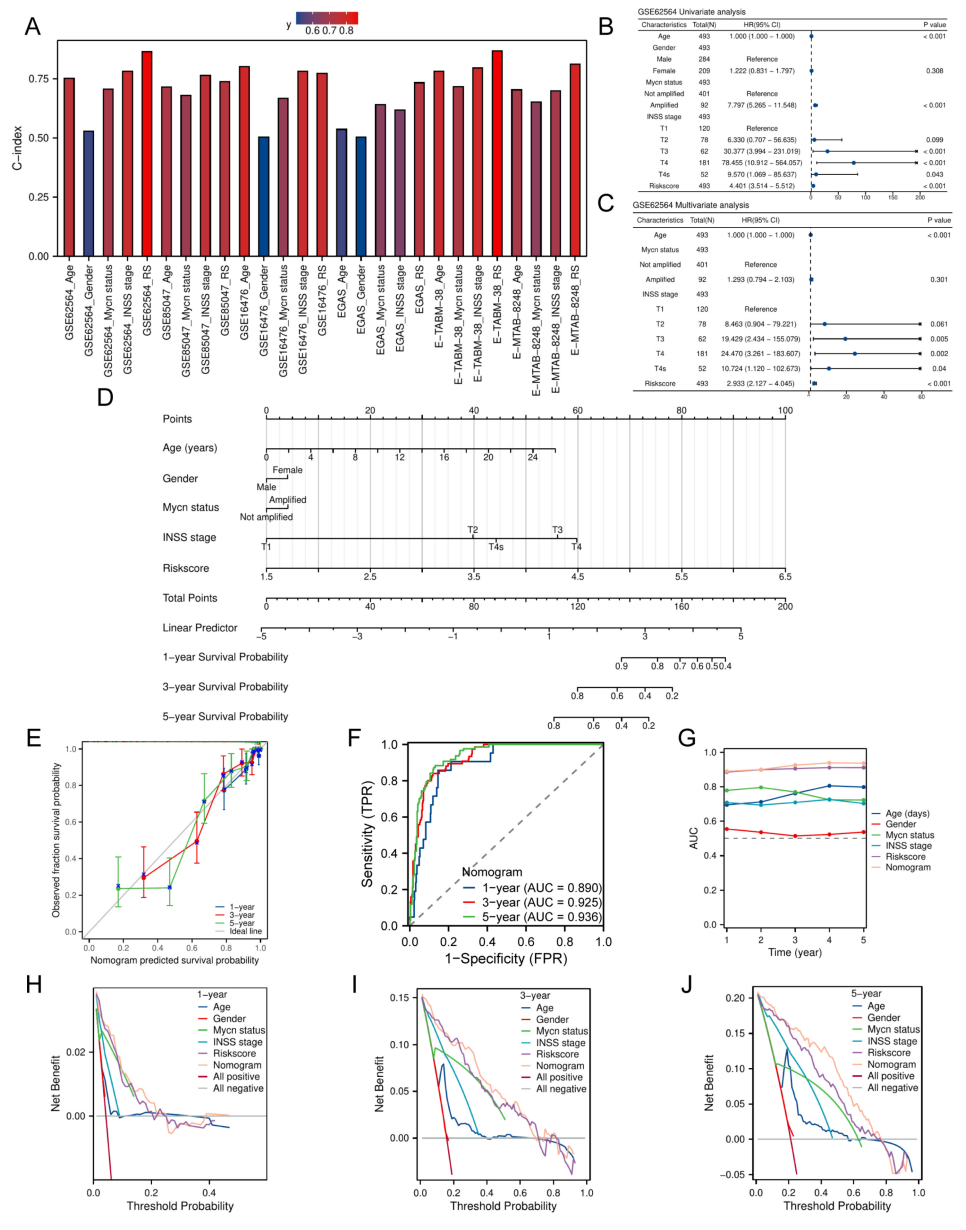
Nomogram construction in the training cohort. **(A)** Comparison of C-index for the risk score and different clinical characteristics across training cohort and five external validation cohorts. **(B)** Univariate Cox regression forest plot including various clinical characteristics and risk score. **(C)** Multivariate Cox regression forest plot including various clinical characteristics and risk score. **(D)** Nomogram established to predict overall survival probability based on clinical characteristics and TRDEGs risk score.** (E)** Calibration plot for the nomogram. **(F)** The time-dependent ROC curves of the nomogram model for the 1-, 3- and 5-year OS. **(G)** ROC curve analysis showing the AUC of age, gender, *MYCN* status, INSS stage, risk score and nomogram.** (H-J)** DCA curves of the nomogram for 1-, 3- , and 5- year OS.

We calculated the clinical factors (age, gender, *MYCN* status, INSS stage, etc.) and risk score C-index for NB patients and compared the differences in the C-index across each cohort. The results showed that every cohort had up to at least three clinical characteristics and the risk score, which was calculated based on the TRDEGs signature's coefficients, demonstrated superior predictive accuracy as measured by C-index compared to any other clinical characteristics (**Figure 4A**). To establish the independent prognostic capacity of this risk score and clinical parameters, we performed univariate and multivariate Cox analyses within all cohorts respectively. We included five NB clinical characteristics - age, gender, *MYCN* status, INSS stage, and risk score - in Cox regression analyses within the training cohort. Univariate Cox regression indicated non-significant associations for gender and INSS stage 2 (*P* > 0.05), whereas INSS stages 3, 4, and 4S, age, *MYCN* status and risk score (HR > 1, *P* < 0.05; **Figure 4B**) exhibited significant prognostic relevance. In subsequent multivariate analysis, though *MYCN* status no longer showed statistical significance (*P* > 0.05), the risk score still maintained its significance as a strong independent prognostic indicator (HR = 2.933, *P* < 0.001; **Figure 4C**). External validation across multiple cohorts consistently corroborated these findings (**Figure S3A-J**). Furthermore, we constructed a predictive nomogram model that integrates multiple clinical characteristics, including the TRDEGs signature's risk score, to predict OS at 1, 3, and 5 years for NB patients (**Figure 4D**), and the nomogram C-index was 0.887 (95% CI 0.875-0.900). As illustrated by the calibration curve, the nomogram demonstrated excellent predictive performance (**Figure 4E**). Our model's high accuracy was further underscored by the AUCs, which attained 0.890, 0.925, and 0.936 for the 1-, 3-, and 5-year intervals, respectively (**Figure 4F**). In addition, ROC analysis further confirmed that the nomogram incorporating other clinical factors demonstrated superior predictive performance (**Figure 4G**). The nomogram provided considerable net benefit in predicting survival outcomes at 1, 3, and 5 years, as confirmed by DCA (**Figure 4H-J**). Collectively, these findings affirmed that our TRDEGs risk score-based model can serve as an independent and accurate prognostic indicator.

### Immune characteristic related to TRDEGs signature

**Figure 5:**
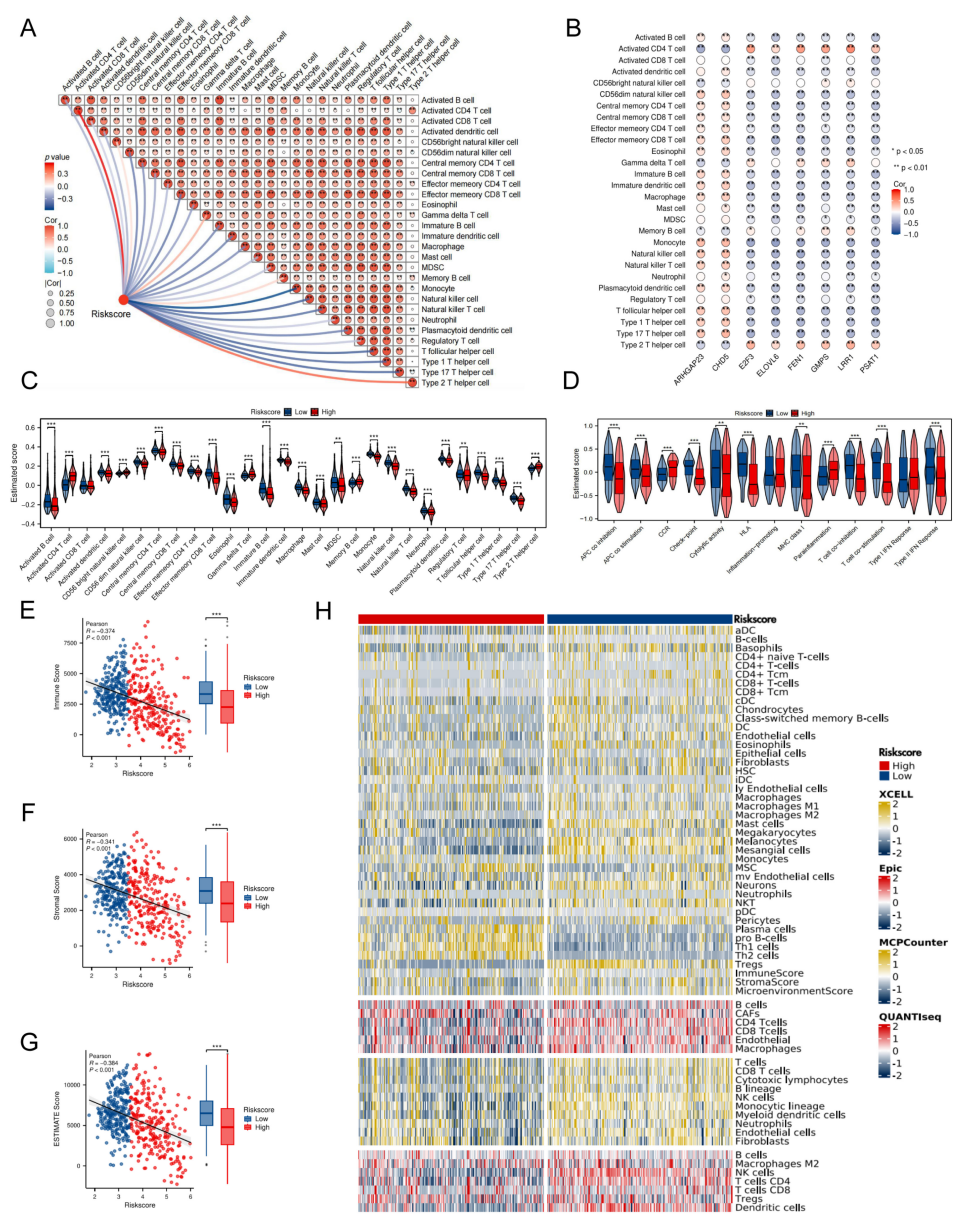
Characterization of tumor microenvironment between two risk groups. **(A)** Correlation heatmap presenting the association between risk score and the relative abundance of immune cells.** (B)** Correlation heatmap showing the relationship between 8 prognostic genes and 28 immune cells infiltration. **(C)** The immune cell infiltration between two risk groups.** (D)** Comparison of immune function in two different groups. **(E-G)** Scatter and box plots showing differences in Immune, Stromal, and Estimate scores between the TRDEG-based risk groups. **(H)** Heatmap of immune cell infiltration between two risk groups using multiple computational algorithms.

Initial ssGSEA analysis of the training cohorts revealed significant inverse associations between the TRDEGs risk score and most signature hub genes (excluding *ARHGAP23* and *CHD5*) and the infiltration levels of diverse tumor-infiltrating immune cells (**Figure 5A, B**). Consequently, high-risk patients exhibited a significant decrease in various types of anti-tumor immune cells. This impairment encompassed both components of innate immunity, such as eosinophils, macrophages, monocytes, natural killer (NK) cells, neutrophils, and plasmacytoid dendritic cells, as well as elements of adaptive immunity, comprising activated B cells, type 1 T helper cells, type 17 T helper cells, and T follicular helper cells (**Figure 5C**). Further analysis of immune functional activity demonstrated broad downregulation in the high-risk group; however, CCR activity, parainflammation, and Type I IFN response were elevated (**Figure 5D**). Consistently, Stromal, Immune, and Estimate scores were markedly reduced in the high-risk group relative to low-risk patients, reflecting a disrupted tumor immune microenvironment (TIME) and a poorer prognostic outcome (**Figure 5E-G**). Validating these findings, complementary analyses employing EPIC, MCPCOUNTER, QUANTISEQ, and XCELL algorithms yielded similar results (**Figure 5H**).

### TRDEG-based signature predicts NB response to immunotherapy

**Figure 6:**
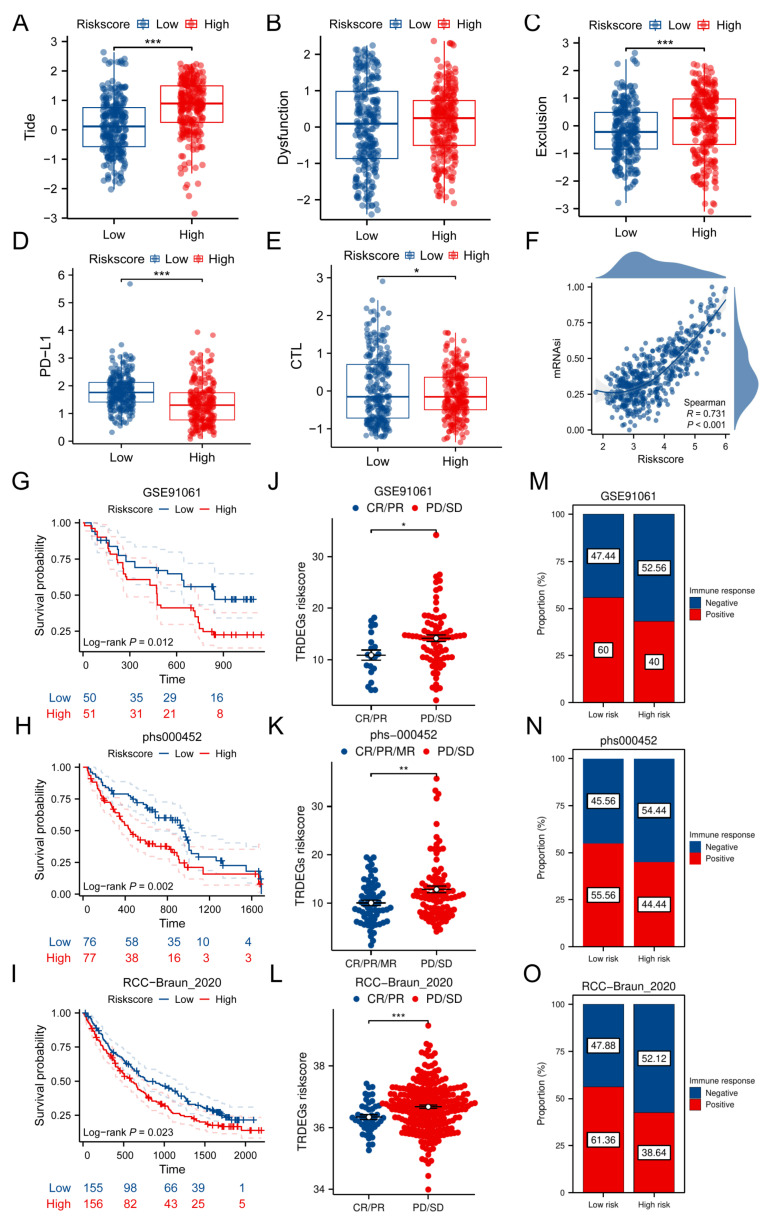
TRDEG-based risk score predicts response of immunotherapy. **(A-E)** Boxplots exhibiting the significant difference in TIDE, Dysfunction, Exclusion, *PD-L1* and CTL score. **(F)** Correlation analysis between model risk score and mRNAsi index. **(G-I)** Kaplan-Meier curves demonstrating a significant variation in survival rate between risk groups in the GSE91061, phs000452, and RCC-Braun_2020.** (J-L)** Wilcoxon rank-sum test of TRDEGs risk score variation in the GSE91061, phs000452, and RCC-Braun_2020.** (M-O)** The stacked histogram illustrating immunotherapy response variation across risk groups in GSE91061, phs000452, and RCC-Braun_2020. *P < 0.05, ** P < 0.01, *** P < 0.001.

We firstly used TIDE algorithms, CTL score and expression pattern of *PD-L1* to assess the immunotherapy reaction using transcriptomic information from patients with NB. Though no notable disparities were observed in dysfunction score, the results indicated that the high-risk group was associated with elevated TIDE and decreased CTL, *PD-L1* levels. This suggests that patients belonging to the low-risk group might experience greater advantages from immunotherapy (**Figure 6A-E**). Secondly, our results indicated a significant positive correlation of TRDEGs risk scores with tumor mRNAsi. The GSE62564 dataset scatter plot validated a strong linear association (R = 0.731, *P* < 0.001), where the fitted trend line clearly underscored this direct relationship (**Figure 6F**). Furthermore, building on prior analysis, we computed TRDEGs risk scores in three immunotherapy cohorts: GSE91061 (anti-PD-1), phs000452 (anti-PD-1), and RCC-Braun_2020 (anti-PD-1 + EVEROLIMUS). Notably, low-risk patients in each cohort exhibited significantly improved survival outcomes and were more responsive to immunotherapy (**Figure 6G-O**).

### Forecasting chemotherapeutic response in NB using TRDEG-based risk score

**Figure 7:**
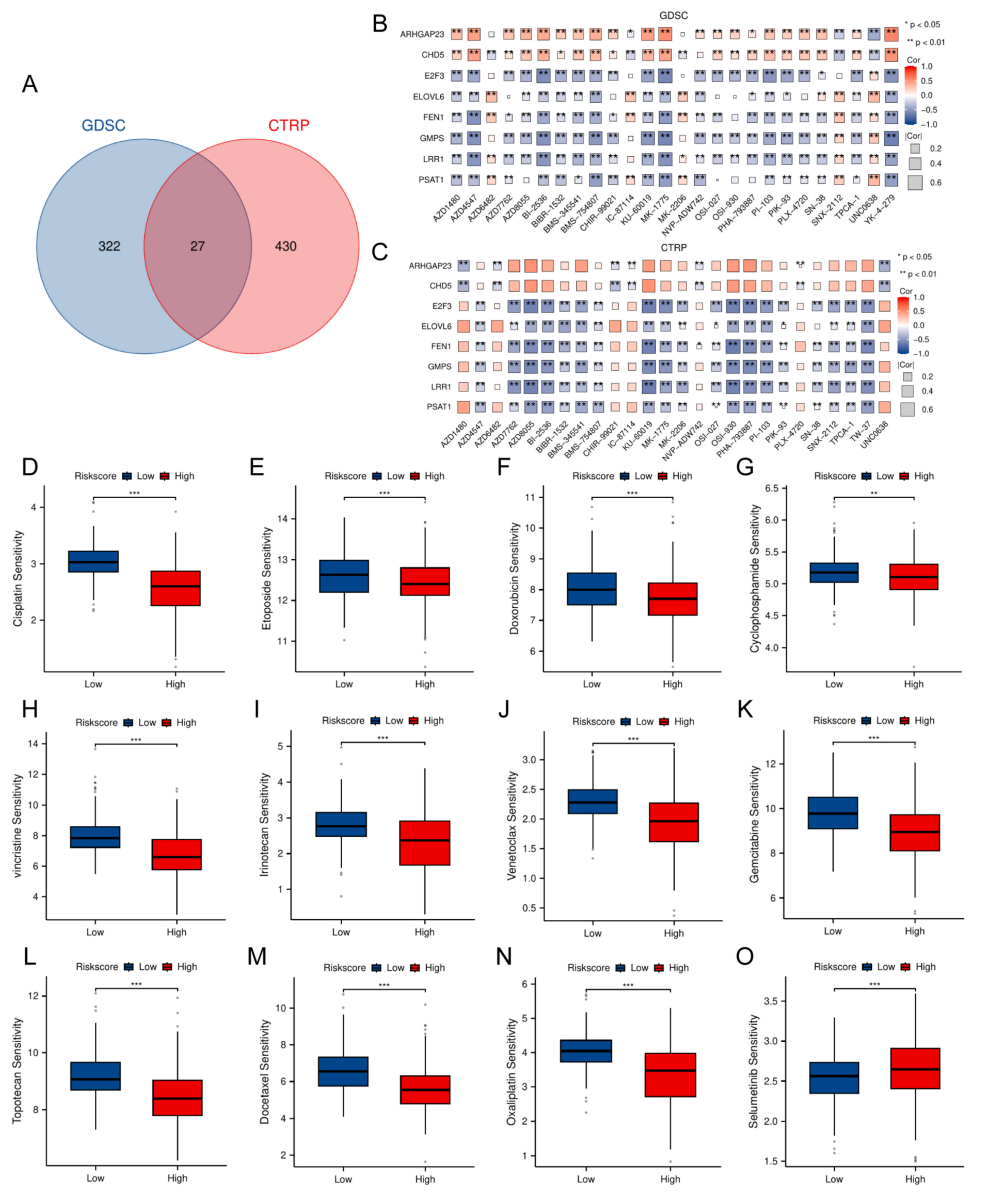
Association between the TRDEG-based risk score and drug sensitivity in NB. **(A)** The Venn diagram shows a total of 27 potential therapeutic agents from GDSC and CTRP. **(B-C)** Matrix showing correlation between 27 potential drugs sensitivity and mRNA expression of the prognostic model genes. **(D-O)** Evaluation of NB related clinical chemotherapy drugs sensitivity between two risk groups. *P < 0.05, ** P < 0.01, *** P < 0.001.

To identify potential therapeutic agents associated to the TRDEGs risk score, we performed drug sensitivity analysis using the "oncoPredict" R package. Differential drug response between risk groups was assessed via Wilcoxon rank-sum test (*P* < 0.05), identifying 349 significantly sensitive compounds in GDSC (from 442 tested) and 457 in CTRP (from 545 tested) (**Figure 7A**). Subsequent analysis focused on identifying drug intersections within the two drug databases. Correlation between the TRDEG signature genes and drug sensitivity was evaluated via Spearman's method. While antagonistic interactions were observed for some gene-drug pairs (e.g., *ARHGAP23* and *CHD5*), synergistic relationships were identified for others, including *E2F3, ELOVL6, FEN1, GMPS, LRR1*, and *PSAT1* (**Figure 7B, C**). To evaluate the TRDEG signature's utility for guiding chemotherapy, we applied the TRDEGs risk score to guide chemotherapeutic selection for NB in clinical practice. Exploring in the NB related chemotherapy drugs, most agents, excluding selumetinib, demonstrated enhanced efficacy in the low-risk group compared to the high-risk group (**Figure 7D-O**). These results highlight the feasibility of tailoring chemotherapy regimens to specific NB risk subgroups, supporting precision oncology approaches.

### Analysis of single-cell RNA-seq data of TRDEG-based signature in NB

**Figure 8:**
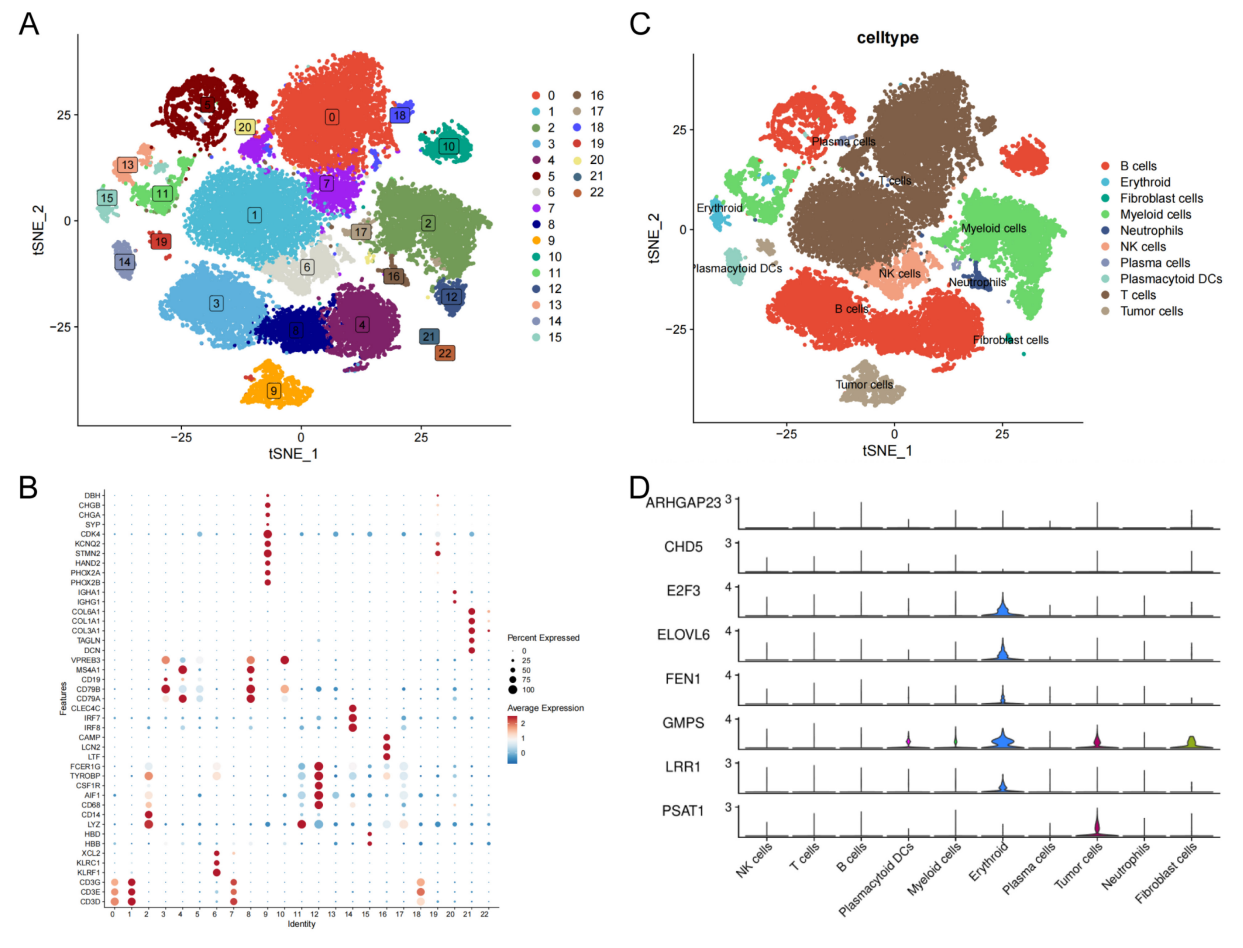
Single-cell transcriptomic landscape of TRDEG-based genes in the NB tumor microenvironment. **(A)** t-SNE plot illustrating the distribution of 23 cell clusters. **(B)** Dot plot showing canonical marker genes used to annotate individual clusters. **(C)** Annotated t-SNE clustering of cell types.** (D)** Violin plots depicting the expression patterns of TRDEG-based prognostic genes across various cell populations.

This study further validated the expression level of TRDEGs signature's hub genes at the single cell level. For single-cell transcriptomic sequencing data derived from GSE220946 (38,026 cells of 15 NB patients), initial processing included quality control, normalization, and batch effect removal. After tSNE-based dimensionality reduction organized cells into 23 clusters, cell-type annotation using established markers revealed 10 distinct populations, including B cells (*CD79A, CD79B, CD19, MS4A1, VPREB3*), erythroid (*HBD, HBB*), fibroblast cells (*DCN, TAGLN, COL3A1, COL1A1, COL6A1*), myeloid cells (*LYZ, CD14, CD68, AIF1, CSF1R, TYROBP, FCER1G*), neutrophils (*LTF, LCN2, CAMP*), NK cells (*KLRF1, KLRC1, XCL2*), plasma cells (*IGHG1, IGHA1*), plasmacytoid DCs (*IRF7, IRF8, CLEC4C*), T cells (*CD3D, CD3E, CD3G*) and tumor cells (*PHOX2B, PHOX2A, HAND2, STMN2, KCNQ2, CDK4, SYP, CHGA, CHGB, DBH*) (**Figure 8A-C**). We discovered that *E2F3, ELOVL6, FEN1* and *LRR1* were primarily expressed in erythroid; *GMPS* was mainly expressed in plasmacytoid DCs, myeloid cells, erythroid, fibroblast cells and tumor cells; *PSAT1* was predominantly expressed in tumor cells. However, there were no significant differences among cells on the expression pattern of *ARHGAP23* and *CHD5* (**Figure 8D**).

### *PSAT1* serves as a predominant NB-associated gene within the TRDEG model

**Figure 9:**
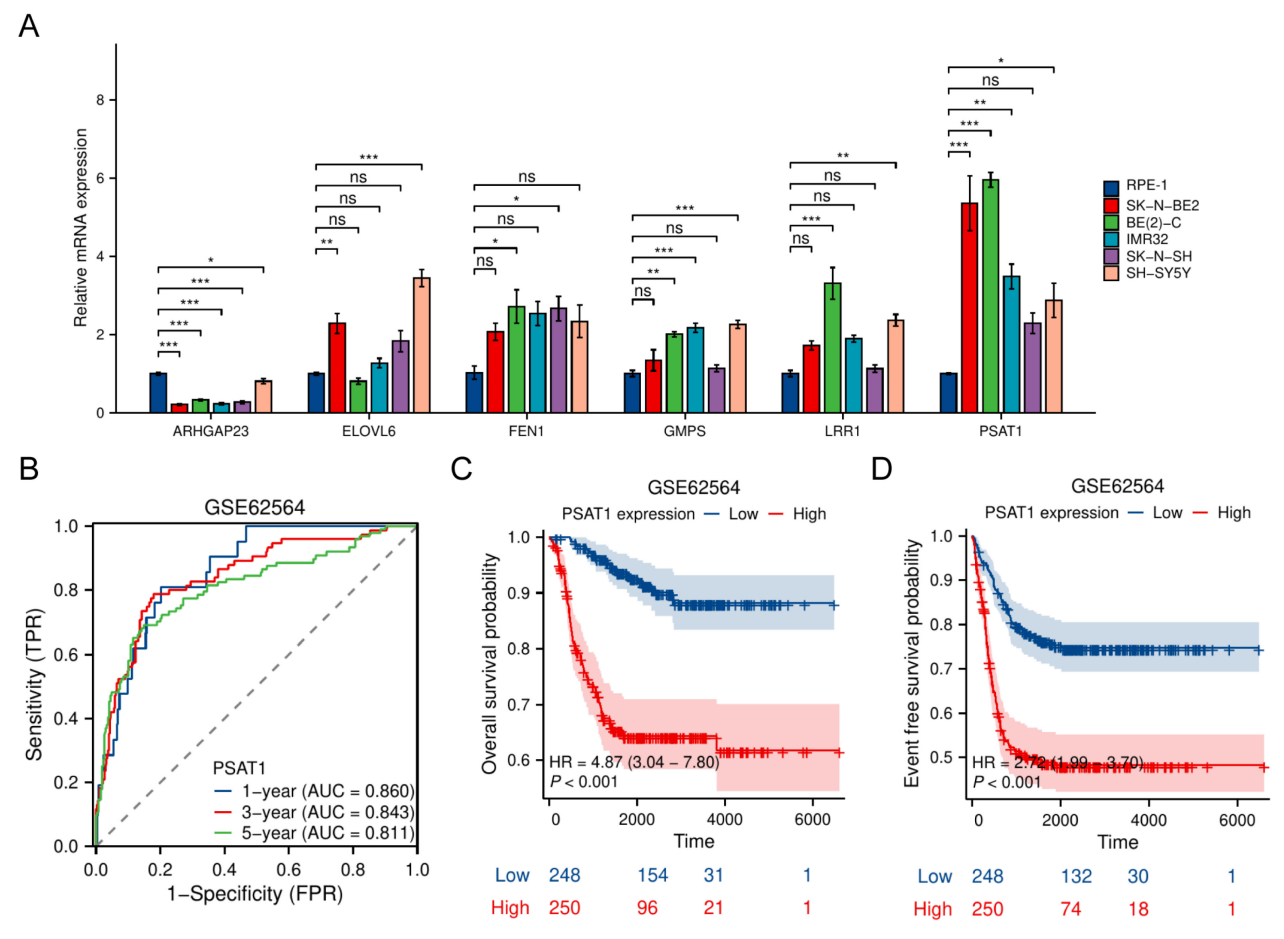
*PSAT1* serves as a NB-dependent gene within the TRDEG-based model.** (A)** Relative mRNA expression of representative TRGs in normal RPE-1 cells and various NB cells. **(B)** Time-dependent ROC curves of *PSAT1* in the GSE62564 cohort predicting 1-, 3-, and 5-year survival. **(C-D)** Kaplan-Meier survival curves comparing OS and EFS between high and low* PSAT1* expression groups.

To experimentally validate the expression and function of the TRDEGs risk score, we measured expression of six risk model genes via quantitative RT-PCR in NB cell lines. The other two genes including *CHD5* and *E2F3* have been demonstrated by other researchers 21-24. Most NB cell lines exhibited markedly elevated *PSAT1* expression compared to the normal RPE-1 cells, with the highest levels observed in SK-N-BE2 and BE(2)-C. Conversely, *ARHGAP23* expression was substantially higher in RPE-1 cells (**Figure 9A**). Importantly, scRNA-seq data led to the identification of *PSAT1* as a representative gene within the TRDEG signature (**Figure 8**). Time-dependent ROC curve analysis demonstrated that *PSAT1* possessed robust predictive accuracy for 1-, 3-, and 5-year survival, with AUCs of 0.860, 0.843, and 0.811, respectively (**Figure 9B**). Kaplan-Meier survival analyses further revealed that patients with high *PSAT1* expression had significantly poorer OS (**Figure 9C**) and event-free survival (**Figure 9D**) compared with those with low expression.

### *PSAT1* acts as a key oncogenic driver in NB progression

**Figure 10:**
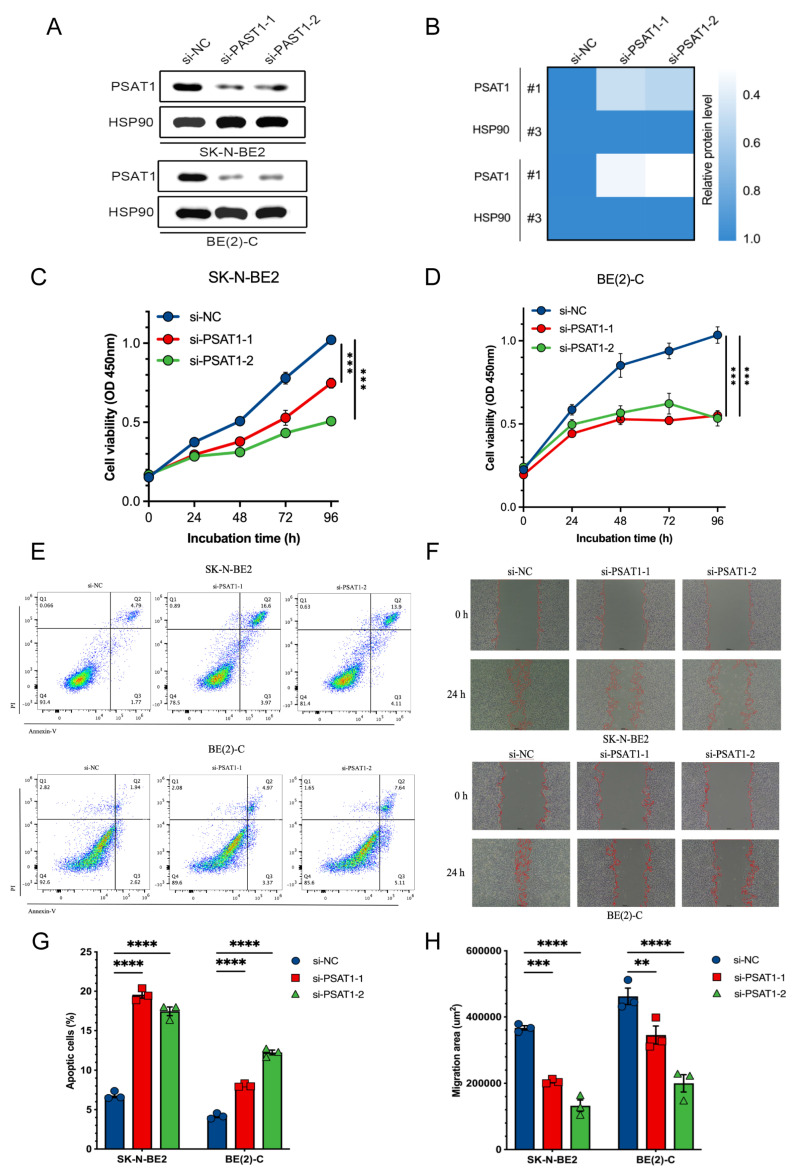
Functional validation of *PSAT1* silencing in NB cells. **(A)** Immunoblotting analysis showing *PSAT1* silenced in SK-N-BE2 and BE(2)-C cells using two independent siRNAs. **(B)** Heatmap showing relative protein levels of *PSAT1* normalized to HSP90 following siRNA-mediated silencing.** (C-D)** Cell viability curves showing reduced proliferation after *PSAT1* silenced in SK-N-BE2 and BE(2)-C cells. **(E)** Flow cytometry analysis of apoptosis in SK-N-BE2 (upper panel) and BE(2)-C (lower panel) cells. **(F)** Wound-healing images showing that inhibited cell migration after *PSAT1* silenced in SK-N-BE2 (upper panel) and BE(2)-C (lower panel) cells. **(G-H)** Quantification of apoptotic cell percentages and migration areas. *P < 0.05, ** P < 0.01, *** P < 0.001.

We first performed *PSAT1* knockdown to verify its experimental relevance using siRNAs in both SK-N-BE2 and BE(2)-C cell lines, as determined by immunoblotting (**Figure 10A, B**). Then CCK-8 analysis assessed proliferative responses to *PSAT1* knockdown in NB cells and both siRNAs significantly suppressed proliferation across SK-N-BE2 and BE(2)-C cell lines (**Figure 10C, D**). In addition, increased apoptosis was identified following *PSAT1* knockdown in NB cells using Annexin V/PI staining (**Figure 10E, G**). A 24-h wound healing assay also confirmed that *PSAT1* inhibition effectively attenuated the migration ability of NB cells (**Figure 10F, H**). Collectively, these results demonstrate that *PSAT1* downregulation inhibits NB cell proliferation and migration while inducing apoptosis, highlighting its oncogenic function in NB.

## Discussion

Responsible for approximately 15% of childhood cancer fatalities, NB persists as the most frequent extracranial solid tumor in children 25, 26. Pronounced molecular heterogeneity drives divergent clinical outcomes and frequently limits therapeutic efficacy, necessitating novel biomarkers and refined molecular subtyping systems to guide personalized treatment decisions 27. Bioinformatics strategies show particular promise for these investigations. Telomeres—specialized nucleoprotein complexes at chromosomal termini—maintain genomic stability. Key telomere-associated genes include telomerase components, telomeric repeats, telomerase-activating receptors, and telomerase inhibitors 28, 29. Dysregulation of these elements correlates strongly with oncogenesis in multiple malignancies: renal carcinoma, lung adenocarcinoma, pancreatic cancer, and glioma 7, 10, 12, 30. Although TMMs constitute a fundamental cancer hallmark enabling limitless replication, TRGs' broader influence on NB's TIME and clinical progression remains inadequately characterized. We elucidate TRGs' contribution to NB development and prognosis through advanced bioinformatics analysis of transcriptomic datasets. Bulk and scRNA-seq integration established and validated a TRG-based prognostic risk model. The risk score demonstrated robust correlations with OS, clinicopathological parameters, immune infiltration patterns, and chemotherapy/immunotherapy responses. These findings establish both theoretical foundations and novel predictive tools for precision management of this challenging pediatric malignancy.

To achieve this objective, a set of 103 TRDEGs was identified at the intersection of DEGs, TRGs from the TelNet database and WGCNA-derived module genes exhibiting strong positive correlation with COG high-risk NB. Using the expression profiles of these selected genes, we stratified NB patients into two discrete molecular subtypes: cluster 1 and cluster 2. This clustering not only differentiated the patient populations but also indicated divergent prognostic outcomes, with Cluster 1 demonstrating poorer prognoses enriched in pathways related to cell proliferation and metabolism, such as the cell cycle and P53 signaling pathways, in contrast to Cluster 2 which was associated with immune regulatory processes, including Cytokine-Cytokine Receptor Interaction and antigen processing. Moreover, most of the top 25 TRDEGs were significantly increased in cluster 1 comparing cluster 2. These findings suggest that immune function in cluster 1 may be suppressed relative to that in cluster 2, potentially mediated by the specific roles of these dysregulated telomere-related molecules.

Moreover, we developed a prognostic risk score model for NB. Through univariate Cox regression and LASSO-Cox regression analysis, eight TRDEGs (*ARHGAP23, CHD5, E2F3, ELOVL6, FEN1, GMPS, LRR1*, and *PSAT1*) were identified as core components of this model.* CHD5*, the fifth member of the nine-protein CHD family, is located on chromosome 1p36. This region is frequently deleted in high-risk NB, and the subsequent low expression of *CHD5* is strongly associated with stage 4 NB 21. Diverse functional assays demonstrated that forced *CHD5* expression in NB cell lines harboring 1p loss significantly impaired key metastatic processes, including anchorage-independent growth, endothelial adhesion, invasion, and migration in vitro 21, 22. As a key regulator of G1/S transition in the cell cycle, the *E2F3* belongs to the E2F family. This family modulates numerous oncogenic processes—including DNA transcription, apoptosis, DNA damage repair, senescence, and autophagy 31. Additionally, *E2F3* has been reported to interact synergistically with *MYCN* and its downregulation significantly induces apoptosis and differentiation 32, 33. Thus, *CHD5* is established as a tumor suppressor in NB, actively inhibiting tumor growth and progression, while *E2F3* is documented as a cancer promoter, exacerbating NB development.

While the functions of the remaining core genes in NB are rarely explored, existing literature shows that these genes are strongly associated with tumor progression, invasion, and metastasis. *ARHGAP23* is a member of the ARH gene family and functions as encoding a GTPase in the Rho family. It acts not only as a tumor suppressor by inhibiting RhoA/Rac1-driven invasion and metastasis, but also contributes to oncogenesis through genetic rearrangements like the *ARHGAP23::FER* fusion 34, 35. We hypothesize that *ARHGAP23* may act as an anti-oncogene based on its markedly reduced mRNA levels in NB cell lines, but its protective mechanism remains unclear; Within *FGFR3*-driven bladder tumors, *ELOVL6*-driven lipid composition preserves mitochondrial function, thereby enabling cell proliferation and oncogenic progression 36; The knockdown of *FEN1* was shown to attenuate AKT/mTOR signaling activation, inhibit pancreatic cell progression in vitro, and suppress tumor growth in vivo in mouse xenografts 37; *GMPS*, an enzyme integral to purine biosynthesis that utilizes glutamine as a nitrogen donor, has emerged as a critical metabolic node in tumorigenesis. For instance, elevated *GMPS* mRNA levels have been found to correlate with higher Gleason scores and to predict reduced overall and disease/progression-free survival rates in prostate cancer patients 38. Importantly, Pharmacological inhibition of *GMPS* with angustmycin A, a potent and selective antagonist, led to the marked attenuation of *PD-L1* expression and oncogenic progression in hepatocellular carcinoma (HCC), concurrently enhancing susceptibility to anti-CTLA-4 immunotherapy 39. These findings in other malignancies suggest that *GMPS* may also play a significant role in NB progression and influence the tumor's response to immunotherapy; Moreover, *LRR1* plays a pivotal role in maintaining genomic stability by facilitating the disassembly of the replisome complex during DNA replication. Disruption of *LRR1* activity, particularly through the CRL2LRR1 enzyme complex, results in the activation of an *ATR*-mediated G2/M checkpoint, causing cell cycle arrest and mitotic blockage 40; *PSAT1*, which is a pivotal enzyme in the serine synthesis pathway, functions as a central node in cancer metabolism and progression, with roles spanning from enzymatic support to non-canonical signaling in migration, metastasis, and therapy resistance 41, 42. To confirm the model's validity, *PSAT1* was selected as a primary regulator among TRDEGs, and subsequent cellular assays demonstrated its role in driving NB cell proliferation and migration. Together, these results underscore the prognostic relevance of these genes and their potential clinical utility in evaluating NB progression and outcomes.

Beyond its well-characterized enzymatic role as the rate-limiting enzyme in the serine synthesis pathway (SSP), *PSAT1* functions as a core constituent of the telomere-related prognostic signature established in our study. Notably, *PSAT1* is officially annotated as a telomere-associated gene in the TelNet database, yet direct experimental evidence linking *PSAT1* to telomere homeostasis regulation in human malignancies has yet to be reported. To address this clear research gap, we outline evidence-based mechanistic hypotheses to delineate the indirect but functionally coherent links between *PSAT1* and telomere biology in NB, focusing specifically on two core metabolic regulatory axes. The first axis centers on *PSAT1*'s essential role in supplying nucleotide precursors for telomere maintenance: as both the rate-limiting enzyme for de novo serine biosynthesis and a key modulator of intracellular one-carbon metabolism, *PSAT1* drives serine production, which serves as the primary cellular source of one-carbon units required for de novo purine and pyrimidine synthesis 43,44. This metabolic cascade provides key upstream metabolic support for telomeric DNA replication and length maintenance 41, processes mediated by either telomerase activation or the alternative lengthening of telomeres (ALT) pathway in high-risk NB 14-17. The second, equally critical axis focuses on *PSAT1*'s ability to preserve telomere structural integrity through redox homeostasis regulation. Telomeric DNA is defined by G-rich repeat sequences, a structural feature that confers extreme vulnerability to oxidative damage from reactive oxygen species (ROS), and this form of oxidative damage is repeatedly demonstrated in prior studies as a core driver of non-replicative, accelerated telomere shortening 45, 46. Through the SSP, *PSAT1* generates glycine, an essential precursor for the production of glutathione (GSH), the cell's primary endogenous antioxidant. By sustaining intracellular redox balance and scavenging excess ROS, *PSAT1* limits oxidative damage to telomeric DNA, and in turn, indirectly protects the structural integrity of telomeres 47, 48. Importantly, we explicitly note that all the associations outlined above are evidence-based hypotheses derived from existing published research, and the direct causal relationship between *PSAT1* and telomere homeostasis in NB still requires systematic functional validation in future studies.

TIME encompasses the structural, metabolic, and cellular components—including stromal, immune, and malignant cells—within the host tissue of a tumor. Crosstalk between tumor cells and immune components within the TIME critically regulates disease progression processes like tumor growth, metastasis, and drug resistance, while also influencing anti-tumor immunity 49, 50. Besides, recent studies have revealed that TRGs significantly associate with immune cell infiltration levels, the composition of TIME, and predicted or potential response to immunotherapy across various cancers 51-53. Given this, elucidating the correlation between the TIME and TRDEGs risk score is essential. Our findings revealed distinct immune landscapes and differential immune cell infiltration between high- and low-risk NB patients. Low-risk NB patients manifested an immunologically active ("hot") microenvironment, characterized by increased infiltration of activated B cells, DCs, CD8⁺ T cells, NK cells, macrophages, and type 1 T helper (Th1) cells, whereas high-risk patients exhibited an immunologically suppressed ("cold") profile dominated by type 2 T helper (Th2) cell accumulation. Prior studies indicate Th1 cells are significant within the TIME, primarily through their activation of CD8+ T cells to drive antitumor responses 54. NK cells critically control tumor cell destruction and metastasis, functioning collaboratively within the TIME (55). By secreting immunoglobulins, B cells support humoral responses and tumor suppression (50). Additionally, CD56dim NK cells and macrophages execute vital anticancer roles across malignancies 56, 57. In contrast to these favorable roles, Fridman's review highlighted that the infiltration of Th2 cells correlates with unfavorable prognoses in ovarian, pancreatic, and gastric cancers, findings that align with our observations 58. Moreover, the low-risk NB patients exhibited higher ESTIMATE, CTL, and *PD-L1* scores alongside a lower TIDE score compared to high-risk patients, indicating a more favorable response to immunologic treatment. To validate these results, we analyzed the TRDEGs risk score in multiple immunotherapy cohorts, with analyses consistently demonstrating prolonged survival and significantly elevated treatment response rates in low-risk patients relative to high-risk counterparts. These results indicate that immunotherapy response in NB could be modified by TRGs through immunocyte efficacy regulation. Understanding these immune dynamics in conjunction with TRGs expression patterns may provide critical insights into optimizing treatment regimens that leverage the patient's immune response while addressing the inherent tumor biology of NB.

This study has several limitations that should be considered. Our findings rely largely on publicly accessible transcriptomic datasets. Although these datasets offer breadth, they likely fail to capture the full pathophysiological spectrum of NB in clinical settings. A particular constraint is the lack of large-scale clinical validations for the TRDEGs identified, which limits opportunities to explore their functional mechanisms and could affect how broadly applicable our results are. For our in vitro experiments, we only applied two *MYCN*-amplified NB cells, which included SK-N-BE(2) and its clonal subline BE(2)-C. The lack of parallel functional tests in other *MYCN*-amplified and *MYCN*-not amplified NB cell models may further limit the widely applicability of our conclusions across different NB subtypes. Moreover, we did not validate our findings in xenograft models. This means we cannot fully recapitulate NB's in vivo tumor microenvironment and physiological growth, so the clinical translation potential of our findings need further exploration. Our single-cell analyses face dual constraints: limited sample sizes inherent to available scRNA-seq datasets, and unavoidable subjectivity during cellular subtype annotation—factors that possibly influenced interpretive accuracy. For immunotherapy response prediction, we used immunotherapy cohorts from melanoma and renal cell carcinoma, and substantial heterogeneity in the tumor immune microenvironment across different cancer types is an inherent limitation of this cross-cancer analysis. Additionally, the modest size of available retrospective cohorts further restricts the robustness of our findings, and future prospective validation in dedicated NB immunotherapy cohorts is essential to confirm its clinical applicability. Overall, further studies with larger clinical samples and in-depth functional assays are needed to verify the clinical translation potential of our findings.

In conclusion, through integrated bioinformatics analysis, we identify that TRGs play a pivotal role in shaping NB prognosis and treatment response. The validated TRDEG signature provides a foundation for individual risk assessment and future development of precision therapies and immunotherapeutic strategies. Among these genes, *PSAT1* emerges as a key oncogenic driver.

## Supplementary Material

Supplementary figures.

Supplementary tables.

## Data Availability

All data and results utilized in this study were obtained from the following database: GEO (http://www.ncbi.nlm.nih.gov/geo/, accessed on 1 September 2025), R2 platform (https://hgserver1.amc.nl/cgi-bin/r2/main.cgi, accessed on 1 September 2025) and TIGER portal (http://tiger.canceromics.org/, accessed on 25 August 2025). Other data relevant to this study are available upon reasonable request from the corresponding author.

## Abbreviations

ALT: Alternative Lengthening of Telomeres
*ARHGAP23*: Rho GTPase Activating Protein 23
AUC: Area Under the Curve
BP: Biological Processes
BSA: Bovine Serum Albumin
CCK-8: Cell Counting Kit-8
CC: Cellular Components
CDF: Cumulative Distribution Function
*CHD5*: Chromodomain Helicase DNA Binding Protein 5
COG: Children's Oncology Group
CR: Complete Response
CTRP: Cancer Therapeutics Response Portal
DCA: Decision Curve Analysis
DEGs: Differentially Expressed Genes
*E2F3*: E2F Transcription Factor 3
EFS: Event-Free Survival
*ELOVL6*: ELOVL Fatty Acid Elongase 6
EPIC: Estimation of the Proportions of Immune and Cancer cells
ESTIMATE: Estimation of Stromal and Immune cells in Malignant Tumor tissues using Expression data
*FEN1*: Flap Endonuclease 1
GDSC: Genomics of Drug Sensitivity in Cancer
GEO: Gene Expression Omnibus
*GMPS*: Guanine Monphosphate Synthase
GO: Gene Ontology
GSEA: Gene Set Enrichment Analysis
HCC: Hepatocellular Carcinoma
HR: Hazard Ratio
INSS: International Neuroblastoma Staging System
KEGG: Kyoto Encyclopedia of Genes and Genomes
LASSO: Least Absolute Shrinkage and Selection Operator
*LRR1*: Leucine-Rich Repeat protein 1
LRS: LASSO Risk Score
MCPCOUNTER: Microenvironment Cell Population Counter
MF: Molecular Functions
mRNAsi: mRNA Stemness Index
NB: Neuroblastoma
OS: Overall Survival
PCA: Principal Component Analysis
PD: Progressive Disease
PR: Partial Response
*PSAT1*: Phosphoserine Aminotransferase 1
QUANTISEQ: Quantification of the Tumor Immune Contexture from RNA-seq data
ROC: Receiver Operating Characteristic
SD: Stable Disease
TIDE: Tumor Immune Dysfunction and Exclusion
TIME: Tumor Immune Microenvironment
TMMs: Telomere Maintenance Mechanisms
TRGs: Telomere-Related Genes
TRDEGs: Telomere-Related Differentially Expressed Genes
UMAP: Uniform Manifold Approximation and Projection
WGCNA: Weighted Gene Co-expression Network Analysis
