# Supplementary material for: Integrative Bulk and Single-Cell Transcriptome Profiling of Telomere-Related Genes Reveals a Robust Prognostic Signature and Immunotherapeutic Landscape in Neuroblastoma
Source: J Cancer. 2026 May 18;17(5):1050–72. doi: 10.7150/jca.129718 (PMC13189846; doi:10.7150/jca.129718)
Supplement: Supplementary file 1 — Supplementary figures. [file jcav17p1050s1.pdf]

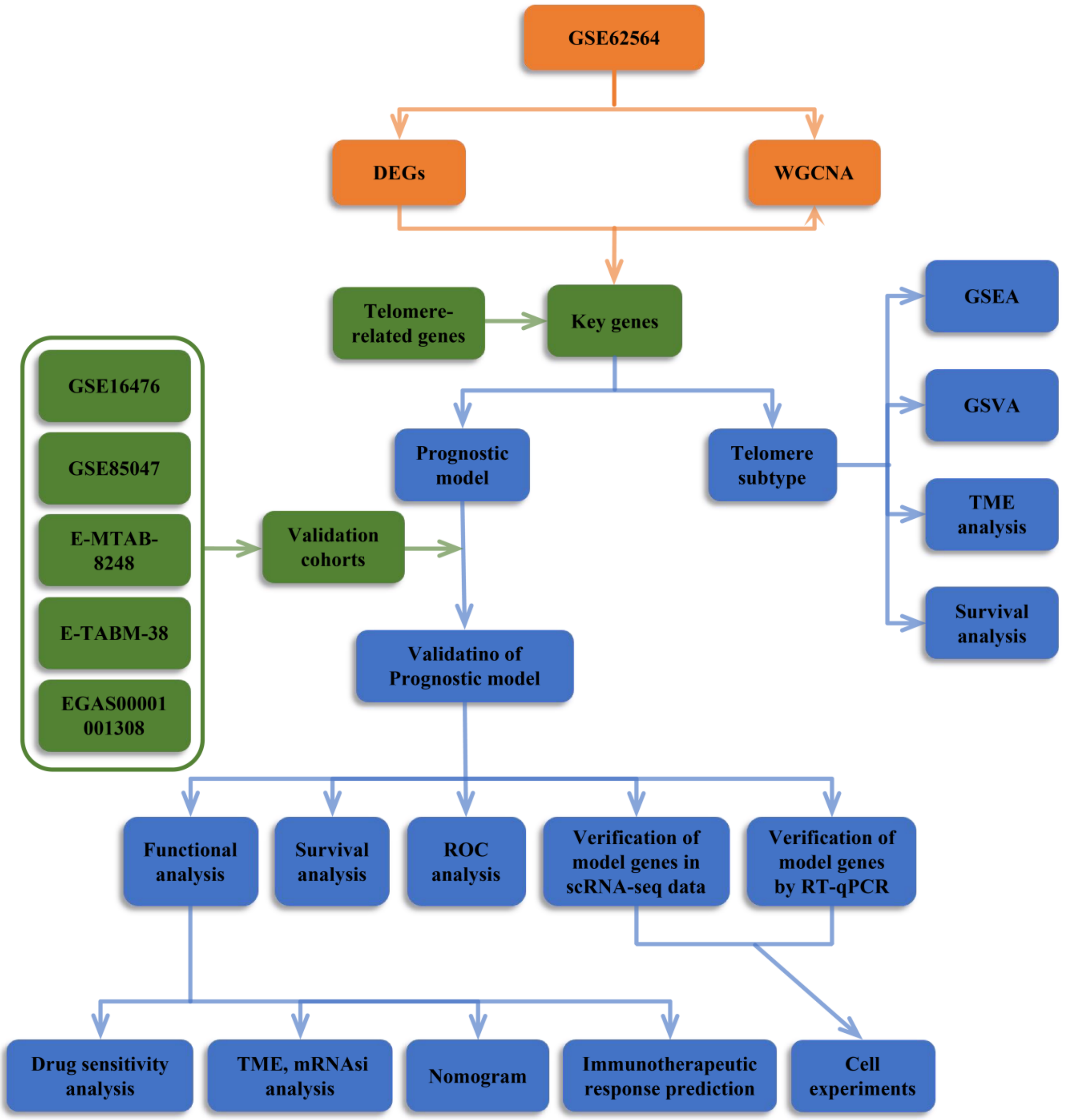

Supplementary Figure 1 Workflow of study.

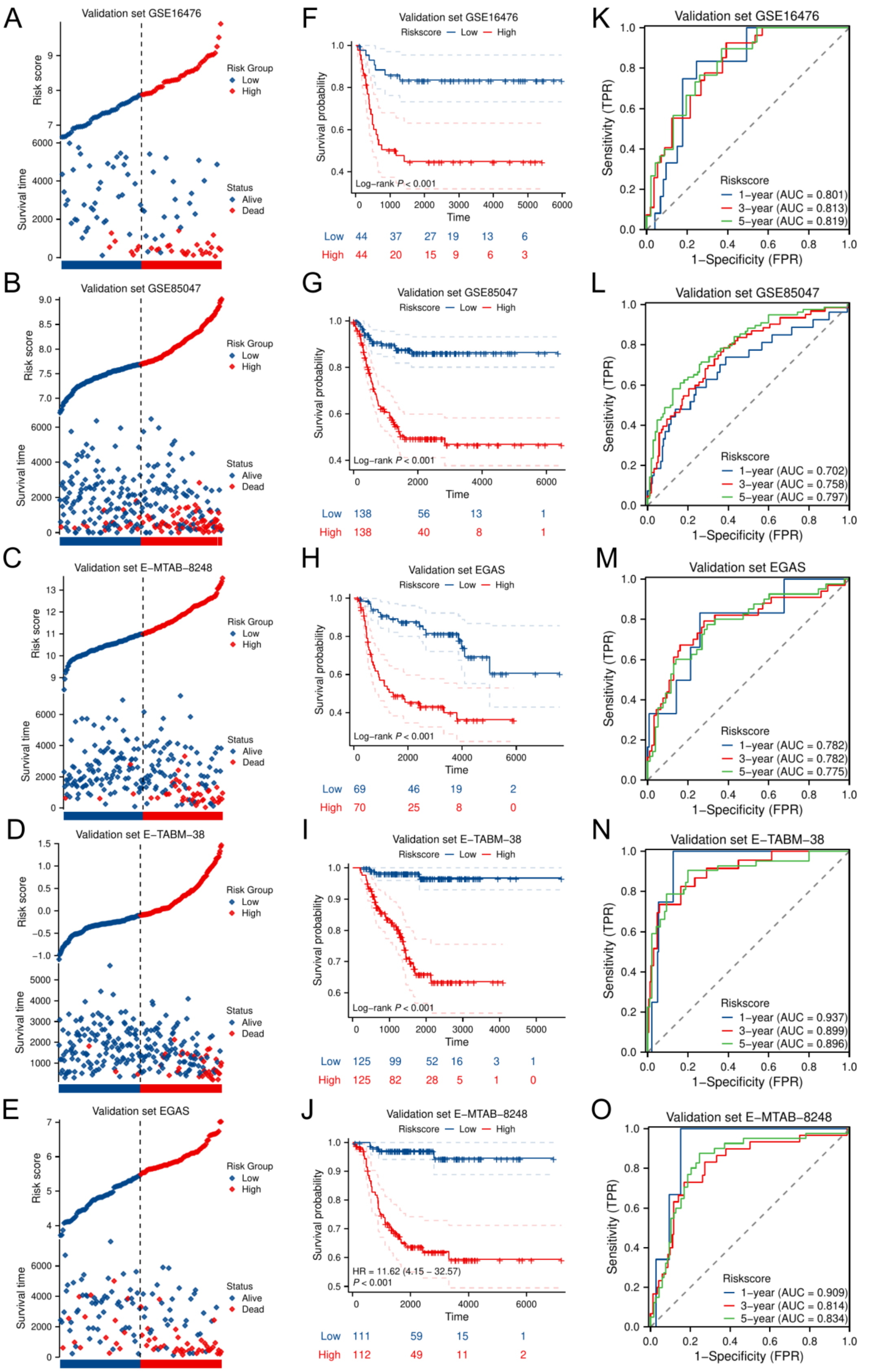

Supplementary Figure 2 External validation of prognostic risk model in multiple cohorts. (**A-E)** Risk curve as well as scatter plots of GSE16476, GSE85047, E-MTAB-8248, E-TABM-38

and EGAS. (**F-J)** Kaplan-Meier curve of GSE16476, GSE85047, E-MTAB-8248, E-TABM-38 and EGAS. (**K-O)** Time-dependent ROC curve of GSE16476, GSE85047, E-MTAB-8248, E-TABM-38 and EGAS.

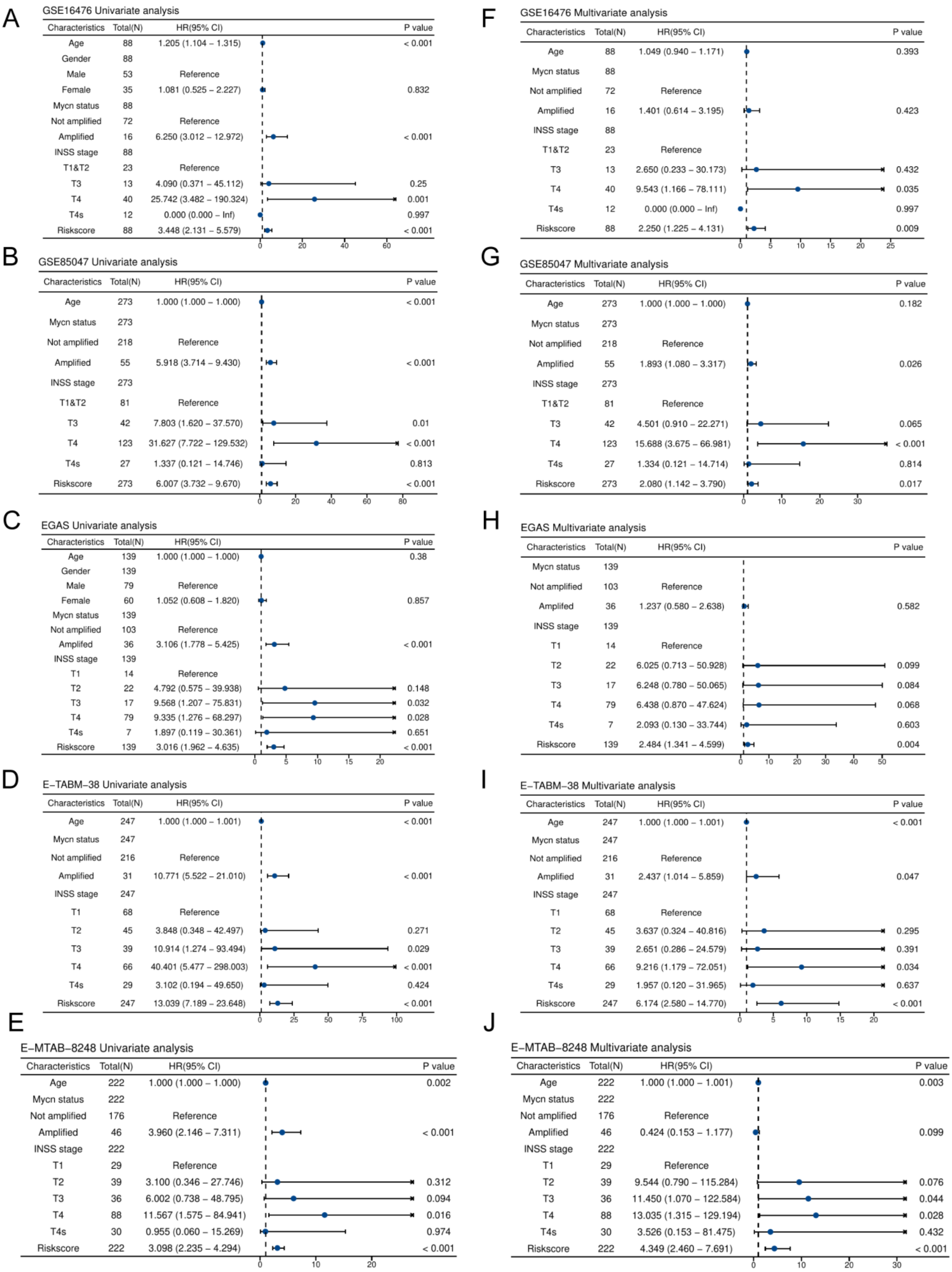

Supplementary Figure 3 TRDEGs risk score is identified as an independent risk factor for neuroblastoma in multiple cohorts. (**A-E**) Independent prognosis-univariate Cox forest plot in

GSE16476, GSE85047, E-MTAB-8248, E-TABM-38 and EGAS. (**F-J)** GSE16476, GSE85047, E-MTAB-8248, E-TABM-38 and EGAS.
